# Inhibition of demethylase by IOX1 modulates chromatin accessibility to enhance NSCLC radiation sensitivity through attenuated PIF1

**DOI:** 10.1038/s41419-023-06346-2

**Published:** 2023-12-12

**Authors:** Qian Li, Kexin Qin, Yushan Tian, Biao Chen, Guoping Zhao, Shengmin Xu, Lijun Wu

**Affiliations:** 1 School of Environmental Science and Optoelectronic Technology, University of Science and Technology of China, Hefei, Anhui 230026 PR China; 2 Information Materials and Intelligent Sensing Laboratory of Anhui Province, Institutes of Physical Science and Information Technology, Anhui University, Hefei, Anhui 230601 PR China; 3 China National Tobacco Quality Supervision and Test Center, Zhengzhou, Henan 450001 PR China

**Keywords:** Radiotherapy, Radiotherapy

## Abstract

Chromatin accessibility is a critical determinant of gene transcriptional expression and regulated by histones modification. However, the potential for manipulating chromatin accessibility to regulate radiation sensitivity remains unclear. Our findings demonstrated that the histone demethylase inhibitor, 5-carboxy-8-hydroxyquinoline (IOX1), could enhance the radiosensitivity of non-small cell lung cancer (NSCLC) in vitro and in vivo. Mechanistically, IOX1 treatment reduced chromatin accessibility in the promoter region of DNA damage repair genes, leading to decreased DNA repair efficiency and elevated DNA damage induced by γ irradiation. Notably, IOX1 treatment significantly reduced both chromatin accessibility and the transcription of phytochrome interacting factor 1 (PIF1), a key player in telomere maintenance. Inhibition of PIF1 delayed radiation-induced DNA and telomeric DNA damage repair, as well as increased radiosensitivity of NSCLC in vitro and in vivo. Further study indicated that the above process was regulated by a reduction of transcription factor myc-associated zinc finger protein (MAZ) binding to the distal intergenic region of the PIF1. Taken together, IOX1-mediated demethylase inactivation reduced chromatin accessibility, leading to elevated telomere damage which is partly due to PIF1 inhibition, thereby enhancing NSCLC radiosensitivity.

## Introduction

In the eukaryotic nucleus, DNA is tightly associated with histones to package into chromatin. Chromatin accessibility determines the recruitment of effector proteins on the DNA-specific docking sites to control gene expression. Alteration in chromatin state have been associated with many diseases, including several cancers [1, 2]. Since the assay for transposase-accessible chromatin using sequencing (ATAC-seq) was developed to assess genome-wide chromatin accessibility in 2013, numerous studies have shown that aberrant alteration in chromatin accessibility regulated tumorigenesis, metastatic progression, radio/chemo resistance, and putative regulatory elements driving these process [3–5]. The chromatin accessibility landscape of primary human cancers has been generated from 410 tumor samples spanning 23 cancer types, and putative interactions between distal regulatory elements and gene promoters linked to cancer predisposition were predicted [6]. Meanwhile, chromatin accessibility associated with protein-RNA correlation in human cancer has been identified through applying multi-omics profiling of chromatin accessibility, RNA abundance, and protein abundance [7]. More importantly, the dynamics of chromatin accessibility directly correspond to tumor resistance to conventional chemotherapy and radiotherapy. A previous study has shown that drug-tolerant cancer cell subpopulations harbor a repressed chromatin state, which requires an increased level of H3K9me3 over LINE-1 elements [8]. It has been demonstrated that the clinical response to histone deacetylase inhibitors (HDACi) in cutaneous T-cell lymphoma is strongly associated with a concurrent gain in chromatin accessibility [9]. These researches represent rational modulation of chromatin accessibility could enhance the effectiveness of therapies such as chemotherapy and radiotherapy through regulating the foundational framework of gene expression in cancer cells.

It is well known that chromatin accessibility is highly correlated with histone modifications such as methylation, acetylation, and phosphorylation. Emerging evidence indicates that the homeostatic balance between methylation and demethylation, which is regulated by demethylase, plays critical roles in cellular maintenance and response to intra- and extracellular stresses through their influence on chromatin structure. The methylation of H3K36, could regulate the activity of the Rpd3S complex to prevent nucleosome turnover [10]. Additionally, H3K36me3 enhances the affinity of the inhibitory Isw1b chromatin remodeling complex to nucleosome, resulting in a maintenance of chromatin constriction and reduction of chromatin accessibility [11]. Targeting the histone methyltransferase subunit EZH2, which has been found to be highly expressed in lung cancer, results in an increase in chromatin accessibility at regions marked by H3K27me3 [12]. Conversely, histone demethylases are upregulated in certain types of cancer. Specifically, histone lysine demethylases (KDMs) of the Jumonji C domain family (JmjC-KDMs) are activated in hypoxic tumors and associated with tumor aggressiveness and progression [13]. Inhibition of JmjC-KDMs KDM3A and KDM6B could enhance radiosensitivity under hypoxic conditions in esophageal squamous cell carcinoma [14]. Interestingly, histone H3 demethylase inhibition by GSK-J4 promoted the transcriptionally silence of chromatin state through an increase in H3K27 methylation and repressed the genes involving radiation-induced DNA damage repair to enhance radiation antitumor effect in Diffuse Intrinsic Pontine Glioma [15].

Non-small cell lung cancer (NSCLC) is a predominant malignancy representing approximately 80% of total lung cancer cases. This highly aggressive form of cancer contributes to a staggering annual global mortality rate exceeding 1.6 million deaths [16]. Ionizing radiation is routinely used for patients with lung cancer, however, the clinical efficacy is limited due to therapeutic resistance. Since NSCLC was governed by the complex interplay of genes, and the changes of chromatin modification in human lung cancer contributes to tumorigenesis [2, 17], regulation of chromatin accessibility maybe a promising strategy to improve NSCLC radiosensitivity. In this study, we employed 8-hydroxyquinoline-5-carboxylic acid (IOX1), a histone demethylase inhibitor that has demonstrated chemotherapeutic effectiveness against multiple tumor types [18, 19], to explore the fundamental mechanisms of gene expression mediated by alterations in chromatin accessibility in the context of NSCLC response to radiosensitivity. By integrating ATAC-seq with RNA-seq analysis, we revealed that IOX1-induced reduction of chromatin accessibility in the promoter region of DNA damage repair genes, resulting in elevated DNA damage response to irradiation. We further determined that alterations in chromatin accessibility led to a reduction in the expression of helicase phytochrome interacting factor 1 (*PIF1*), which is significantly elevated in NSCLC, was at least partly involved in radiosensitivity of NSCLC. Our findings suggested that inhibition of demethylase modulates chromatin accessibility to improve NSCLC radiosensitivity. Additionally, targeting PIF1 may serve as a promising radiosensitization approach for the treatment of NSCLC.

## Materials and methods

### Cells culture and treatment

The human NSCLC cell lines A549, H1299, H1975, normal human fetal lung fibroblast MRC5, human cervical cancer cell lines HeLa, and HEK293T were all obtained from the Cell Bank of the Chinese Academy of Sciences (Shanghai, China). They were maintained in Dulbecco’s modified Eagle medium (DMEM, Gibco, USA) supplemented with 10% fetal bovine serum (FBS, Biowest, USA) and 1% penicillin/streptomycin and cultured in a 5% CO_2_ incubator at 37 °C. All the cell lines were confirmed by STR genotyping and tested to be free of mycoplasma contamination.

Gamma irradiation was carried out using irradiator (Gamma Service Medical GmbH, Leipzig, Germany) with a ^137^Cs source at a dose rate of approximately 3.27 Gy/min at Hefei Institutes of Physical Science, Chinese Academy of Sciences. The equipment is maintained and calibrated every year by the manufacturer to ensure the precision of the radiation dose.

### Cell transfection

PIF1 short hairpin RNA (shRNA) was generated based on pLKO.1-puro lentiviral vector. This shRNA sequence has been previously shown to be efficiently silenced *PIF1* in the human osteosarcoma cells (U2OS) [20]. PIF1 (NC_000015.10) coding sequence was cloned into pCDH-CMV-MCS-EF1-GFP-puro lentiviral vector. Lentiviral particles were produced in HEK 293 T cells by co-transfection of lentiviral packaging plasmids. Transfections were performed using PEI reagent (Sigma-Aldrich, USA). The lentivirus-infected A549 and H1299 cells were selected with 1.5 μg/ml puromycin for 7 days and stabilized by culturing for 1 weeks. Efficiency of construction was identified by western blot. SiRNA-specific sequences targeting *MAZ* were synthesized by Sangon Biotechnology (Shanghai, China). Transfection of siRNAs into A549 cells was performed using HiperFect reagent (Qiagen, Frankfurt, German) according to the manufacture instructions. Cells were lysed 48 h post-transfection for gene expression analysis. shRNA sequences and siRNAs used in the study are listed in Table S1.

### Apoptosis detection by Annexin V-FITC staining

The Annexin V apoptosis detection kit (BD Biosciences, CA, USA) was used to detect apoptosis. Briefly, cells were seeded in 35-mm dishes and treated with DMSO vehicle or 40 μM IOX1 for 48 h following with or without 4 Gy γ-irradiation. After incubating another 48 h, cells were collected and resuspended in 100 μl binding buffer. Each sample was then incubated with 5 μl Annexin V-FITC and 5 μl PI for 10 min at room temperature in the dark. After that, the samples were added to another 400 μl binding buffer and mixed gently. Flow cytometric analysis was performed immediately with BD FACS Aria^TM^ III (BD Biosciences, USA) to determine the percentages of Annexin V positive cells. Data were analyzed using FlowJo version 7.6 (FlowJo, Ashland, USA).

### Xenograft model

The A549 xenograft experiment has been described previously [21]. Briefly, for IOX1-treated in vivo experiments, 1 × 10^6^ A549 cells were subcutaneously inoculated into the right dorsal of athymic nude female mice (BALB/c Nude) aged 4–6 weeks. When the average tumor size was reached to～50 mm^3^ on the 17th day, they were randomly divided into 2 groups, and no blinding was performed. One group was injected with saline (control group, *n* = 10), the other group was injected with 10 mg/kg IOX1 in saline (treatment group, *n* = 10). The drug treatment was continued once a day for a total of 6 days. During on the 20th and 23th day, half of the mice in both two groups were subjected to γ irradiation (a single 10 Gy dose, twice, *n* = 5). The tumor size (mm^3^) was measured with calipers every 3 days. The maximum diameter (L) and minimum diameter (W) of the tumor tissue were measured by an electric vernier caliper, and the volume was calculated according to the formula: *L* × *W*^*2*^ × 0.52. Mice were euthanized at indicated time points by CO_2_ asphyxiation and the tumors were collected for further analyses. For PIF1-silienced A549 tumor xenograft mouse model, mice bearing A549-shCtrl and A549-shPIF1 tumors were treated with the same procedure but no drug. Among all mice, the SPF facility at Hefei Institutes of Physical Science, Chinese Academy of Sciences (Anhui) was used for breeding and maintenance (Approval number: DWLL-2020-28).

### ATAC-seq and data analysis

A549 cells were treated with DMSO or IOX1 (40 μM) for 48 h, respectively. After that, a total of 1 × 10^6^ cells were collected and nuclei were purified. Subsequently, cell nuclei were fragmented and tagged by Tn5 transposase at 37 °C for 30 min. DNA fragments were then purified by MinElute Reaction Cleanup Kit (Qiagen, Germany). And the library fragments were purified by QIA Quick PCR kits (Qiagen, Germany). Purified DNA was tested by Agilent 2100 (Agilent Technologies, CA, USA). Qualified libraries were sequenced on an Illumina HiSeq 2500 sequencer (Illumina, CA, USA) to obtain the information of open chromatin region fragments. The filtered clean reads were compared with the reference GRCh37/hg19 genome using Bowtie2 (v2.3.2), the BAM file was sorted, and the repeated sequences caused by PCR were labeled. MACS2 (v2.1.2) was used to calculate the sample enrichment area. CHIPseeker was used to identify and annotate the peak-related genes of each sample and obtain the functional information related to the open region. Motif analysis of specific peaks was predicted by HOMER software (v4.11). The edgeR software package was used to analyze the difference between the samples and identify the rich regions with the difference modification between the samples. Peaks with FDR-adjusted *P*-value ≤ 0.05 and fold change ≥|2| were considered as significant enrichment. Integrative Genomics Viewer (IGV) (http://software.broadinstitute.org/software/igv/) was used to visualize and analyze the associated genetic changes in the results.

### ATAC-qPCR analysis

ATAC-qPCR was performed as described previously with minor modifications [22]. Briefly, ATAC-seq libraries in A549 cells were generated according to the method described in the ATAC-seq section. Then, the ATAC-seq library was used in each qPCR reaction as a template. *PIF1* and *TERT* primer sequencing are available in Table S2.

### RNA-seq and data analysis

Total RNA was extracted using Trizol Reagent (Ambion Life Technologies, CA, USA) and quantified by Agilent 2100 Bioanalyzer (Agilent Technologies, CA, USA). First-strand cDNA was synthesized from purified poly(A) mRNA using ProtoScript II Reverse Transcriptase, and the second-strand cDNA was synthesized using Second Strand Synthesis Enzyme Mix. The cDNA libraries were end-prepared and promptly ligated. After being purified and quantified using a Qubit 3.0 fluorometer (Invitrogen, CA, USA), the libraries with different indices were multiplexed and loaded on an Illumina HiSeq instrument (Illumina, CA, USA) according to manufacturer’s instructions. Sequencing was carried out using a 2×150 paired-end (PE) configuration. The data were aligned to reference GRCh37/hg19 genome via software Hisat2 (v2.0.1). Differential expression analysis was analyzed by the edgeR software package, and genes were considered differentially expressed between the groups if FDR-adjusted *P*-value ≤ 0.05 and fold change ≥|2|. GSEA was performed using GSEA software (http://software.broadinstitute.org/gsea/).

### Statistical analysis

Statistical analysis was performed using GraphPad Prism 8 software (GraphPad Software, CA, USA). The data were expressed as mean ± s. d. All cell-based experiments were performed independently 3 times. Animals were randomized into different groups and five mice were used for each group. Two-tailed Student’s *t* test was used to compare differences between two groups and two-way ANOVA test was used to compare differences among multiple groups. A difference was considered to be significant at *P < 0.05, **P < 0.01 or ***P < 0.001.

A description of other experimental details can be found in Supplementary Materials.

## Results

### Demethylase inhibitor IOX1 significantly enhanced the radiosensitivity of NSCLC in vitro and in vivo

**Fig. 1:**
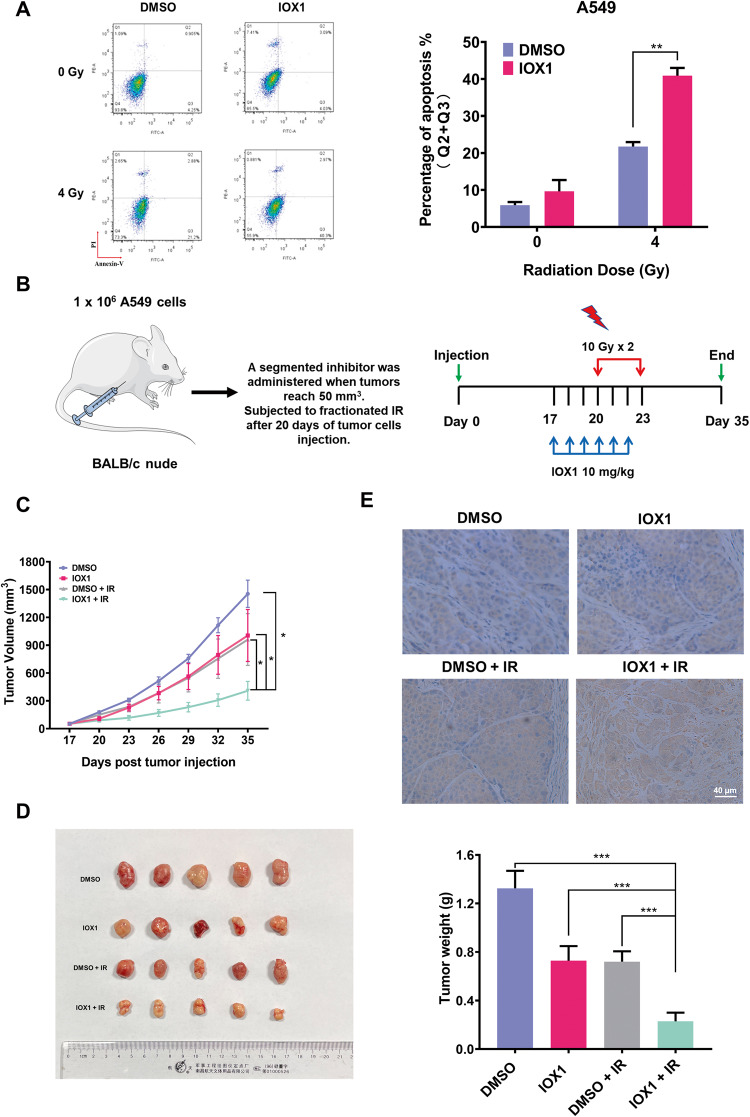
IOX1 enhanced the radiosensitivity of NSCLC in vitro and in vivo. **A** Flow cytometry was used to detect the apoptosis of A549 in the IOX1-treated group and the control group at 48 h after 4 Gy γ-irradiation. Histogram represents (left) the percentage of annexin V^+^PI^−^cells in early apoptosis and annexin V^+^PI^+^ in late apoptosis. **B** Schemes for establishment and treatment of A549 xenograft mouse model. **C** A549 cells (1 × 10^6^) were injected subcutaneously into athymic nude mice. When tumor size reached ~50 mm^3^, mice were pretreated with IOX1 (*n* = 10) or DMSO (*n* = 10), and then divided into two groups which one group received total of 20 Gy irradiation. Tumor growth was followed until tumor size of control reached ～1.5 cm^3^. **D** Representative images of tumors (left) and tumor weights (right) in each group (*n* = 5) at the end of the experiment described in **C**. **E** Cleaved-caspase3 protein expression in each group was verified by immunohistochemical staining. Scale bar, 40 μm. Representative images (*n* = 5) of immunohistochemical staining were assessed. The data are presented as the mean ± SD from three independent experiments. *P*-values were analyzed by Student’s *t* test between two groups and two-way analysis of variance (ANOVA) in multiple groups. *P < 0.05, **P < 0.01, ***P < 0.001.

TCGA database showed that histone demethylases (KDM4A, KDM4B, KDM2A) were overexpressed in NSCLC (Fig. S1A). Studies have suggested that histone demethylases are involved in the regulation of tumor growth [23]. IOX1, a demethylase inhibitor, has been shown to enhance the therapeutic efficacy of cancer treatment by inhibiting demethylase activity. In light of this, we aimed to investigate the potential of IOX1-mediated demethylase inhibition to augment the effectiveness of radiotherapy. To this end, we first used cell apoptosis assay to determine whether the effect of IOX1 treatment on the radiosensitivity of NSCLC. Intriguingly, treatment of 40 μM IOX1 for 48 h, which did not affect cell proliferation of A549 cells (Fig. S1B), was found to significantly elevated γ irradiation-induced apoptosis (~1.88-fold in A549 cells), whereas IOX1 treatment alone did not induce much apoptosis in A549 cells (Fig. 1A). Similar results were observed in another two NSCLC cell lines (H1299 and H1975 cells) and human cervical carcinoma cell line (HeLa cells; Fig. S1C). In addition, IOX1 also remarkably further impaired proliferation as determined by EdU assay both in irradiation-treated A549 and H1299 cells (Fig. S1D, E).

Next, to validate the results of cell experiments, we further investigated whether IOX1 could enhance radiosensitivity of NSCLC in vivo. To this end, a xenotransplantation model of human NSCLC in mice was established in mice using A549 cells. When tumors reached approximately 50 mm^3^ in volume, experiment mice groups received radiation (20 Gy in two fraction) with or without treated with IOX1 (Fig. 1B). As shown in Fig. 1C, D, the IOX1 and radiation combination treatment significantly suppressed the A549 tumor growth compared with the control, IOX1 alone and irradiation alone treatment groups, indicating IOX1 enhanced sensitivity to radiation in an A549 xenograft model. Furthermore, we also analyzed the expression of cleaved caspase 3 (marker of apoptosis) in the tumor tissues isolated from treated and untreated mice, at the endpoint of the experiment. As expected, the level of cleaved-caspase 3 was much higher in combination IOX1-radiation compared with monotherapy (Fig. 1E). Taken together, these results indicated that pretreatment with IOX1, a demethylase inhibitor, significantly enhanced radiosensitivity of NSCLC in vitro and in vivo.

### IOX1 downregulated chromatin accessibility of genes related to DNA damage repair and telomere maintenance

**Fig. 2:**
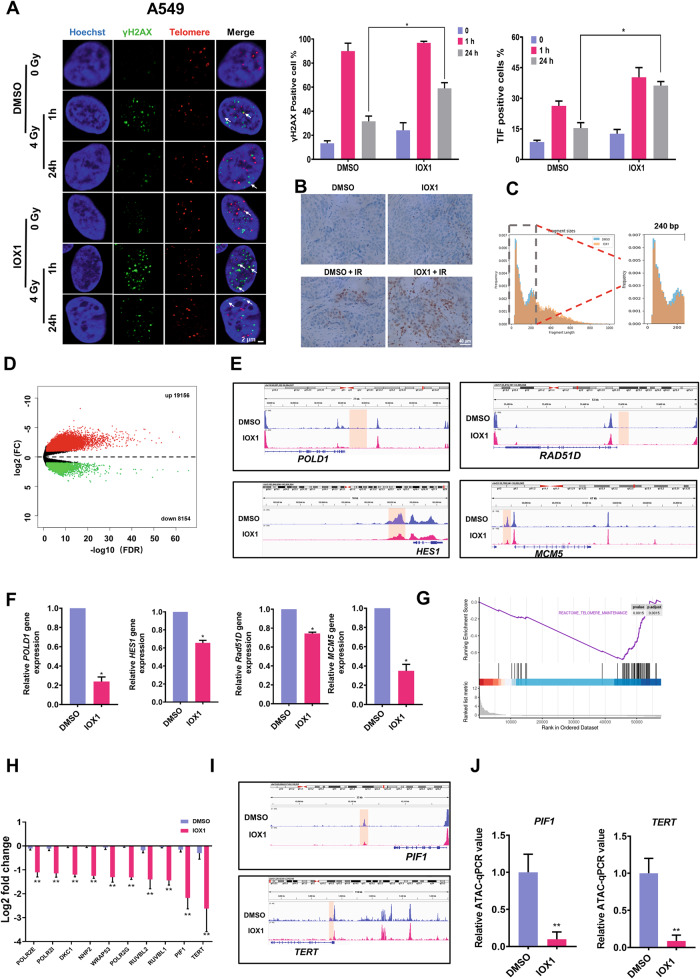
IOX1 decreased chromatin accessibility of genes related to DNA damage repair and telomere maintenance. **A** Immunofluorescence in situ hybridization (FISH) with γH2AX antibody (Green) and telomere PNA probe (Red) was used to detect DNA damage and telomere dysfunction-induced foci (TIFs) in IOX1-treated and DMSO-treated control A549 cells at indicated times after γ-irradiation. White arrows point to telomeric DNA damage. Scale bar, 2 μm. Quantification of γH2AX positive cells (top left) and TIF-positive cells (top right). **B** γH2AX protein expression in each group was verified by immunohistochemical staining. Scale bar, 40 μm. Representative images (*n* = 5) of immunohistochemical staining were assessed. **C** Length distribution of inserted fragments detected by ATAC-seq in A549 cells treated with IOX1 for 48 h. **D** Volcano plot of significant peak changes in chromatin accessibility between control cells and IOX1-treated cells. Red and green dots represent genes significantly upregulated and downregulated (FDR < 0.05) in IOX1 treatment group compared with the control group, respectively. **E** IGV plot for ATAC-seq of indicated DNA repair-related gene loci *(POLD1, HES1, RAD51D, MCM5*). **F** Independent validation of 4 DNA repair-related genes (*POLD1, HES1, RAD51D, MCM5*) by qRT-PCR in IOX1-treated cells. **G** Enrichment plot of telomere maintenance in IOX1-treated A549 cells compared to control cells profiled using gene expression, identified by gene set enrichment analysis (GSEA). **H** Quantitative assessment of telomere maintenance related genes expression at the transcription level in A549 cells treated with IOX1. Signals normalized to control cells. **I** IGV plot for ATAC-seq signals of *PIF1* (top) and *TERT* (bottom) between IOX1-treated cells and control cells. **J** Changes in chromatin accessibility of *PIF1* and *TERT* genes in A549 cells after IOX1 treatment were verified by ATAC-qPCR. The data are presented as the mean ± SD from three independent experiments. *P*-values were analyzed by Student’s *t* test between two groups. *P < 0.05, **P < 0.01.

The major mechanism accounting for radiation-induced cell apoptosis is DNA damage, with double-strand breaks (DSBs) being the most lethal form. It is well known that ionizing radiation kills cancer cells mainly by inflicting DNA damage [24]. Apoptosis in tumor cells exposed to radiation are believed to cause by DNA double-strand breaks (DSBs). However, DSBs can be efficiently repaired by DNA repair mechanisms. Since DNA repair capacity is closely associated with the outcome of radiotherapy, we then assessed DSBs in γ-irradiated cells by γH2AX (marker of DSBs) foci formation assay. As shown in Fig. 2A, when A549 cells were irradiated at 4 Gy, severity of DNA damage was strongly increased at 1 h after irradiation, and there were no significant differences in the percentage of γH2AX positive cells between groups without and with IOX1. Notably, γH2AX foci mostly disappeared at 24 h after irradiation in DMSO control group, implicating effective repair of γ-induced DSBs. However, at 24 h after irradiation, IOX1 treatment delayed repair of γ-induced DNA damage, with the percentage of γH2AX positive cells was ~59% (~1.86-fold compared with control group under the exposure of radiation) in the IOX1-treated group of A549 cells. Telomeres, located at the ends of chromosomes, are the sensitive site of radiation damage in tumor radiotherapy, and particle radiation can cause telomeric DNA damage through direct and indirect effects [25, 26]. As expected, at 24 h after irradiation, approximately 36.23 ± 1.99% of A549 cells showed telomere dysfunction-induced foci (TIFs) at most telomeres, much higher than that of DMSO-treated control cells (~2.35-fold in A549). Similar result was obtained in H1299 cells (Fig. S2). Moreover, the level of γH2AX in A549 xenograft tumor was much higher in combination IOX1-radiation compared with monotherapy (Fig. 2B). These results indicated that IOX1 treatment significantly inhibited DSBs and telomeric DNA damage repair of NSCLC.

Chromatin accessibility provides docking sites for DNA repair factors and other regulatory proteins during DNA damage. Histone modifications often result in altered chromatin accessibility [15]. IOX1 is a known broad-spectrum inhibitor of histone demethylases, including JMJD2/KDM4 family which is acting on the repressive marks H3K9me3 and H3K36me3. Indeed, the protein levels of H3K9me3 and H3K36me3 were significantly increased after IOX1 treatment (Fig. S3). Therefore, we suspected that IOX1 could regulate radiosensitivity of NSCLC by changing the chromatin accessibility. To verify this hypothesis, we performed assays for transposase accessible chromatin with high-throughput sequencing (ATAC-seq). An average of 76.7% mappability and 131 million qualified fragments per sample was obtained (Table S3). ATAC-Seq data from each three repeated DMSO or IOX1-treated samples showed a high correlation (*R* = 0.99, Fig. S4A), indicating that ATAC-Seq was able to reliably and reproducibly measure chromatin accessibility in these samples. We observed reduced chromatin accessibility after IOX1 treatment (Fig. 2C). A total of 27310 differential chromatin areas were detected between the IOX1-treated group and the control group, among which 19,156 were increased and 8154 were decreased (Fig. 2D). And the chromatin accessibility near the transcriptional initiation site in the IOX1-treated group was significantly decreased (Fig. S4B). To understand global gene expression changes of IOX1 treatment, we performed RNA-seq analyses, and identified the following 3627 differentially expressed genes (DEGs) between IOX1-treated A549 cells and vehicle-treated cells: 2381 genes were upregulated and 1246 genes were downregulated (Fig. S4C). Correlation analysis of chromatin accessibility and transcriptome data in different functional regions of the gene showed the highest correlation in the promoter region of the gene (Fig. S4D).

Since IOX1 could elevated radiation-induced DNA damage and delayed the DNA damage repair, we analyzed the chromatin accessibility changes in the promoter regions of genes associated with the damage repair. Genes involved in DNA damage repair, *POLD1*, *HES1*, *RAD51D,* and *MCM5* genes, exhibited a decreased chromatin accessibility near promoter regions (Fig. 2E). In addition, the transcriptional levels of these four genes were confirmed by qRT-PCR (Fig. 2F). Meanwhile, we observed downregulated expression of genes set involved in telomere maintenance in IOX1-treated cells compared to DMSO-treated control cells (gene set enrichment analyses (GSEA) revealed a negative enrichment score for telomere maintenance upon IOX1 treatment) (Fig. 2G). And found that *RTEL1, H2AC14, PRIM1, POLR2E, H2BC13, DKC1, POLR2I, NHP2, WRAPS3, POLR2G, TERT, RUVBL2, RUVBL1, PIF1,* and *H2AC20* genes were significantly decreased at the transcription level after the treatment of IOX1, with *PIF1* and *TERT* genes most substantially downregulated (Fig. 2H). More importantly, ATAC-seq indicated that *PIF1* and *TERT* genes were significantly more inaccessible after IOX1 treatment (Fig. 2I). Meanwhile, chromatin accessibility of *PIF1* and *TERT* genes was further detected by ATAC-qPCR. The results showed that both *PIF1* and *TERT* gene chromatin accessibility were downregulated in A549 cells after IOX1 treatment (Fig. 2J). Taken together, these data showed IOX1 treatment decreased chromatin accessibility of genes related to DNA damage repair and telomere maintenance partially lead to radiosensitivity of NSCLC.

### MAZ-binding motif regulated PIF1 involved in the maintenance of telomere during radiation

**Fig. 3:**
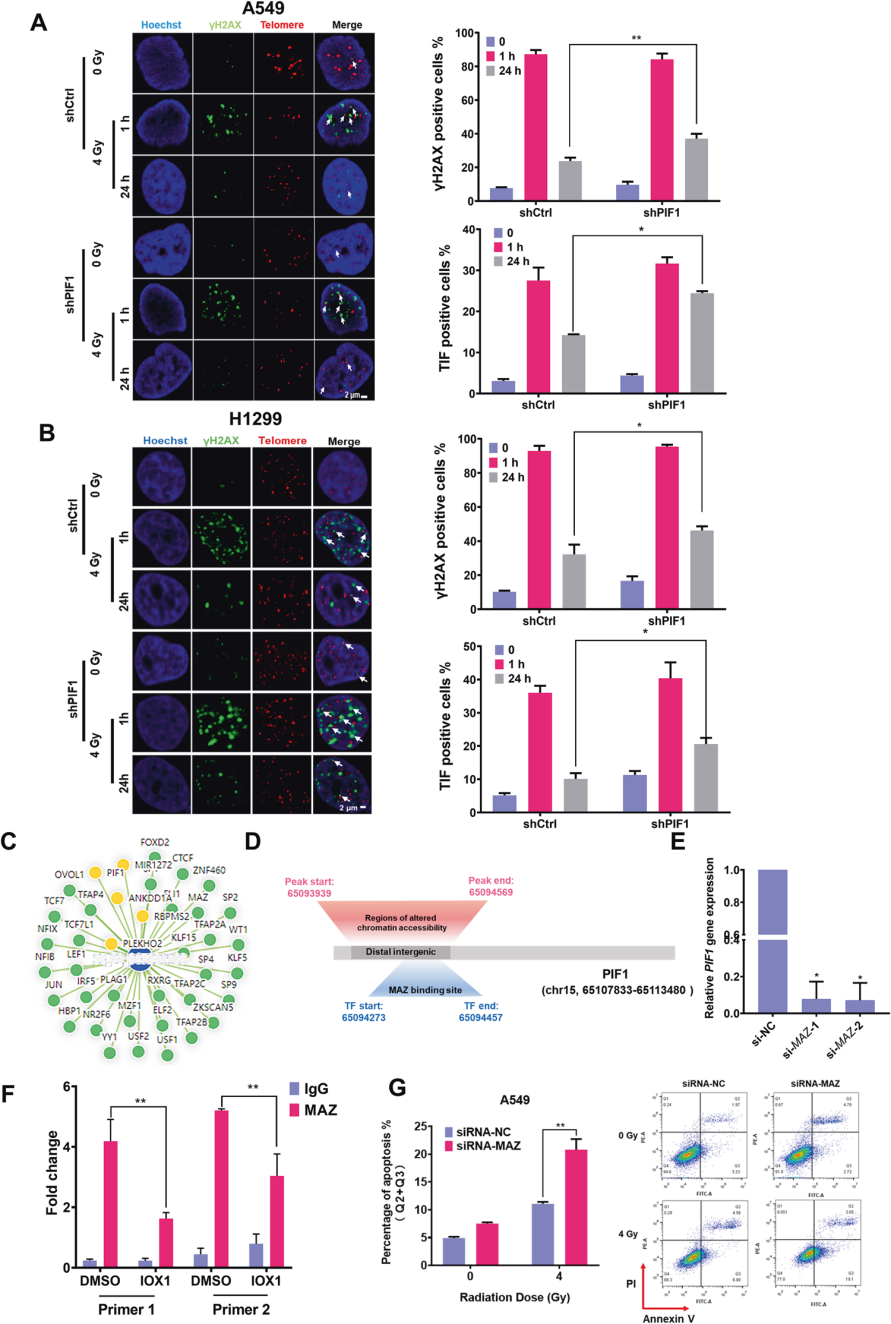
PIF1 involved in the maintenance of telomere during radiation through positive regulation by MAZ. **A** Immunofluorescence in situ hybridization (FISH) with γH2AX antibody (Green) and telomere PNA probe (Red) was used to detect DNA damage and telomere dysfunction-induced foci (TIFs) in PIF1-depleted A549 cells and (**B**) in PIF1-depleted H1299 cells at indicated times after γ-irradiation. White arrows point to telomeric DNA damage. Quantification of γH2AX positive cells (top) and TIF-positive cells (bottom) was shown on the right panel of figure. **C** SEdb software was used to predict transcription factors that bind to enhancer regions. Green circles represent transcription factors, yellow circles represent genes, and blue circle represents enhancer region. **D** Schematic representation of the chromatin accessibility changes in the region of the *PIF1* gene and the potential binding regions of MAZ to *PIF1*. **E** *PIF1* gene expression after interfered with siRNA-*MAZ* in A549 cells was detected by qRT-PCR. **F** Enrichment of MAZ at the distal interstitial region of *PIF1* was confirmed using CUT & Tag-qPCR in A549 cells treated with IOX1 for 48 h. **G** Flow cytometry analysis of the effect of MAZ inhibition on radiation-induced apoptosis in A549 cells. The data are presented as the mean ± SD from three independent experiments. *P*-values were analyzed by Student’s *t* test between two groups and two-way analysis of variance (ANOVA) in multiple groups. *P < 0.05, **P < 0.01.

PIF1 is a helicase which has been described to preferentially regulate G4 structures on the telomere [27]. Notably, we verified that the chromatin accessibility of the *PIF1* gene was reduced and its expression was decreased both in mRNA and protein levels in response to IOX1 treatment (Figs. 2J and S5A). To gain insight into the function of PIF1 on maintenance of telomere during radiation, we generated lentiviral vector expressing PIF1-specific shRNA, which efficiently silenced *PIF1* in A549 and H1299 cell lines (Fig. S5B, C). Knockdown of PIF1 did not affect the DNA damage in A549 and H1299 cell lines. Interestingly, although damage levels subsided at 24 h after 4 Gy irradiation, the percentage of γH2AX positive cells was much higher in shPIF1 group, compared to control at 24 h post-irradiation, indicative of reduced repair of irradiation-induced DNA damage (Fig. 3A, B). To test whether PIF1 is required for telomere maintenance, we examined the effect of depletion of PIF1 on telomere maintenance. Analysis of PIF1‐silenced A549 and H1299 cells at 24 h post‐irradiation revealed a significant increase in γH2AX‐positive TIF formation compared to control group, similar with IOX1 treatment experiments (Fig. 3A, B), underscoring the requirement of PIF1 for telomere maintenance in radiation-treated NSCLC.

Changes in chromatin accessibility affect transcription factors (TF) binding to regulatory elements, thereby regulating the associated gene expression [28]. To identify the key transcription factors that regulate the expression of *PIF1* gene after IOX1 treatment, we further analyzed the ATAC-seq data. It was shown that the alterations in chromatin accessibility of the *PIF1* gene induced by IOX treatment occurred in the distal intergenic region of the gene. Distal intergenic regions potentially serve as enhancer regions, facilitating the binding of transcription factors and thereby contributing to the regulation of gene transcription [29]. As a result, we utilized SEdb 2.0 (http://www.licpathway.net/sedb) to predict potential transcription factors that bind to the enhancer region of the *PIF1* gene (Fig. 3C). It was shown that the region of myc-associated zinc finger protein (MAZ) transcription factor was included in the region displaying changes of *PIF1* gene in chromatin accessibility following IOX1 treatment (Fig. 3D). Additionally, motif enrichment analysis revealed a notable downregulation in MAZ motif enrichment following IOX1 treatment (Fig. S6A). Hence, we proceeded to inhibit *MAZ* gene expression using small interfering RNA to assess its impact on the transcriptional regulation of the *PIF1* gene. As shown in Figs. S6B and 3E, the inhibition of MAZ led to a significant downregulation of *PIF1* gene expression. Meanwhile, the enrichment of MAZ in the distal intergenic region of the *PIF1* gene was markedly reduced after IOX1 treatment, as confirmed by CUT-Tag-qPCR analysis in Fig. 3F. The above results suggested that IOX1 treatment induced a decrease in the binding of MAZ to the distal intergenic region of the *PIF1* gene, thereby suppressing the transcriptional expression of *PIF1*. In addition, WB results showed that MAZ expression was downregulated after IOX1 treatment (Fig. S6C), suggesting that IOX1 regulates *PIF1* transcriptional expression, possibly in part by indirectly affecting MAZ expression. Previous study showed that MAZ promoted chemoresistance in lung adenocarcinoma cells by inhibiting DNA damage [30]. Here, we examined the expression of MAZ in NSCLC and found that MAZ was highly expressed in NSCLC compared with normal lung fibroblasts (Fig. S6D). Meanwhile, inhibition of MAZ further enhanced radiation-induced apoptosis (Fig. 3G). The cell proliferation assay also showed that MAZ inhibition significantly suppressed cell proliferation activity in response to radiation (Fig. S6E, F). These results implied that inhibition of MAZ could enhance the radiosensitivity of NSCLC.

### Inhibition of PIF1 enhanced radiosensitivity of NSCLC in vitro

**Fig. 4:**
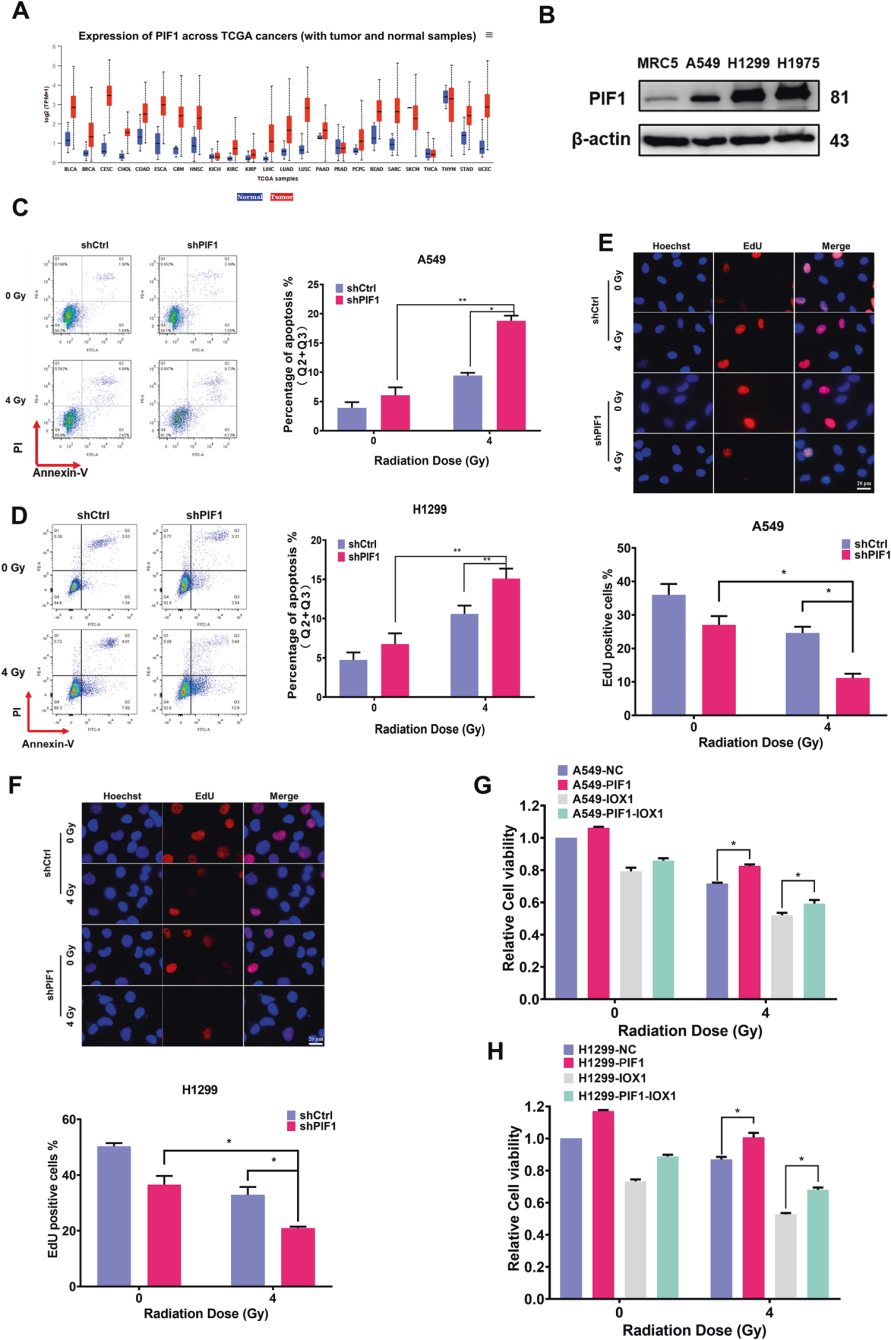
PIF1 modulated radiosensitivity of NSCLC in vitro. **A** TCGA database was used to analyze the expression of *PIF1* gene in 24 cancers and their corresponding normal tissues. **B** Protein expression of PIF1 in three NSCLC cell lines cells (A549, H1299, H1975) and normal human fetal lung fibroblast MRC5 cells was determined by western blot. **C** Apoptosis of *PIF1*-silenced A549 cells and (**D**) H1299 cells was detected by flow cytometry at 48 h post-4 Gy γ-irradiation. **E** Cell proliferation in A549-shPIF1 cells and (**F**) H1299-shPIF1 cells was determined by EdU staining at 48 h post-4 Gy γ-irradiation. Red nuclei indicated EdU incorporation. Scale bar, 20 μm. **G** Cell viability in A549-PIF1 and (**H**) H1299-PIF1 cells treated with IOX1 after 4 Gy γ-irradiation was assessed by CCK8 assay. The data are presented as the mean ± SD from three independent experiments. *P*-values were analyzed by Student’s *t* test between two groups and two-way analysis of variance (ANOVA) in multiple groups. *P < 0.05, **P < 0.01.

The results described above demonstrated the important role of PIF1 in DNA damage repair and telomere maintenance. Moreover, a Pan-cancer analysis of the TCGA database showed that *PIF1* was highly expressed in most tumors compared to the normal tissue (Fig. 4A). In addition, PIF1 protein expression was markedly elevated in A549, H1299, and H1975 NSCLC cell lines than that in normal human fetal lung fibroblast MRC5 cells (Fig. 4B). Thus, we believed that PIF1 may play a critical role in response to radiation for tumors. We therefore examine the ability of PIF1 depletion to enhance radiosensitivity of NSCLC cell lines. As expected, knockdown of PIF1 significantly increased irradiation-induced apoptosis in A549 and H1299 cells (Fig. 4C, D). Cell proliferative activity in PIF1-silenced A549 and H1299 cells was much lower than that in control cell after irradiation (Figs. 4E, F and S7A, B). Importantly, overexpressed the PIF1 gene in these two cell lines (Fig. S7C, D), partially restored the cell proliferation to some extent compared to control cells after irradiation (Fig. 4G, H). These results were also confirmed by EdU assays (Fig. S7E, F). Besides, overexpressed PIF1 could attenuate the radiosensitization effect of IOX1 in A549 and H1299 cells. These results indicated that PIF1 was a crucial gene involved in the regulation of radiosensitivity, and the radiosensitization effect of IOX1 in NSCSC cell lines was at least partially due to the inhibition of PIF1.

### PIF1 silencing significantly increased the radiosensitivity of NSCLC in vivo

**Fig. 5:**
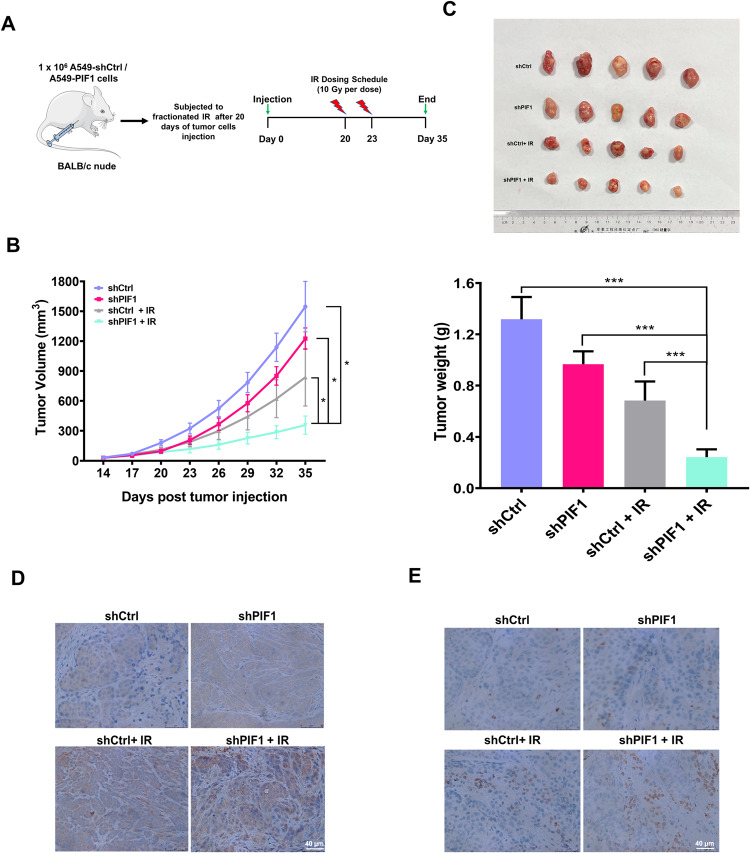
PIF1 silencing increased the radiosensitivity of A549 cell transplanted xenografts tumor model in mice. **A** Schematic illustration of the experiment. **B** Tumor growth curves of A549 tumor-bearing mice. Equal numbers (1 × 10^6^ cells/site) of A549 cells stably expressing either *PIF1* shRNA or control shRNA were injected into female athymic nude mice (5 mice per group). Following irradiation, tumor growth was followed until tumor size in control reached ~1.5 cm^3^. **C** Representative images of tumors (top) and tumor weight (bottom) in each group (*n* = 5) at the end of the experiment described in **B**. **D** Cleaved-caspase3 and (**E**) γH2AX protein expression was verified by immunohistochemical staining. Representative images (*n* = 5) of immunohistochemical staining were assessed. Scale bar, 40 μm. The data are presented as the mean ± SD from three independent experiments. *P*-values were analyzed by Student’s t test between two groups and two-way analysis of variance (ANOVA) in multiple groups. *P < 0.05, **P < 0.01, ***P < 0.001.

Based on the results for regulation of radiosensitivity of PIF1 in vitro, we performed in vivo studies to further validate our findings. PIF1-silenced A549 cells were then inoculated into nude mice, and mice bearing A549-shPIF1 tumors were administered a dose of 2 × 10 Gy γ irradiation (Fig. 5A). As shown in Fig. 5B, C, there was no significant difference in tumor size between the mice inoculated with A549 cells transfected with scrambled shRNA and PIF1 shRNA. Importantly, PIF1 silencing in A549 cells substantially attenuated the growth of the xenograft tumors after subjected to irradiation. Tumors isolated at the endpoint of the study showed significant reduction in tumor weight in the PIF1-silenced group treated with irradiation (Fig. 5C), indicating that inhibition of PIF1 could also enhance the radiosensitivity of NSCLC in vivo. The immunohistochemical analysis revealed that A549 tumor xenografts with silenced PIF1 demonstrated a notable elevation in the protein expression levels of cleaved caspase3 and γH2AX subsequent to irradiation treatment (Fig. 5D, E). We inferred the possibility that the attenuated tumor growth in PIF1-silenced group was due to elevated apoptosis and DNA damage. Taken together, these results provided strong evidence that silencing PIF1 enhanced radiosensitivity of NSCLC in vivo by inhibiting DNA damage repair.

## Discussion

**Fig. 6:**
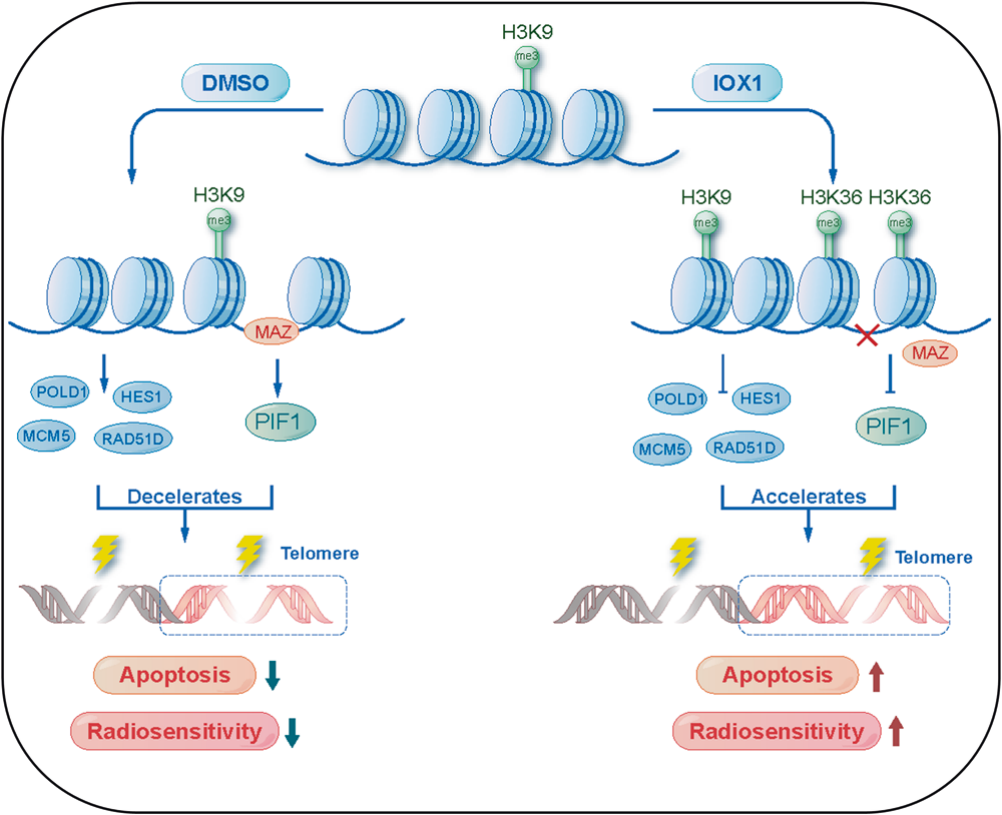
Schematic illustration of the mechanism for IOX1-mediated radiosensitivity in NSCLC. IOX1 treatment caused reduction of chromatin accessibility to the promoter regions of DNA damage repair related genes (*POLD1, HES1, RAD51D, MCM5*) and suppressed their expression. On the other hand, IOX1 decreased the recruitment of MAZ transcription factor at the distal intergenic region of *PIF1* to suppress its transcription. These further lead to an increased radiation-induced DNA and telomere damage, which in turn enhanced the radiosensitivity of NSCLC.

Global changes in the chromatin landscape define gene expression and determine many of biological functions. Previous studies have indicated that some inhibitors could significantly enhanced the radiosensitivity of cancer cells both in vitro and in vivo through altering histone modification states to affect chromatin remodeling and epigenetics [15]. However, the underlying mechanisms are not fully understood. In this study, we investigated the role of IOX1 on radiosensitivity of NSCLC and its potential mechanisms (Fig. 6). IOX1 acts a histone demethylase inhibitor, resulting in elevated levels of H3K9me3 and H3K36me3. This changes in methylation state decrease chromatin accessibility and attenuate the DNA damage and TIFs repair efficiency, thus improving radiation-induced apoptosis. Mechanistically, we show that transcription factor MAZ positively regulates PIF1 at least partially to involve in the IOX1-induced radiosensitivity of NSCLC. This study provides insights into targeting the DNA damage repair and telomere maintenance through chromatin accessibility may be an important avenue for radiotherapy.

IOX1 is a broad-spectrum histone demethylase inhibitor [18, 23, 31], and plays an important role in the regulation of chemosensitivity of tumor cells. Liu et al. found that IOX1 greatly enhanced doxorubicin (DOX)-induced immune-stimulatory immunogenic cell death through inhibition of JMJD1A/β-catenin/P-gp pathway [18]. Another study indicated that IOX1 could also inhibit KDM2A to reduce the proliferation and invasion of bladder cancer cells in vitro and in vivo [23]. However, it remains unclear whether inhibition of demethylases could regulate radiosensitivity by regulation of chromatin accessibility and gene expression. As expected, after IOX1 treatment, an elevated level of H3K9me3 and H3K36me3 in A549 cells were observed. Chromatin accessibility is tightly regulated through multiple mechanisms, including histone modification. Our study showed that chromatin accessibility was significantly decreased near the transcription start site, and a part of DNA damage repair-related genes were both significantly downregulated in the promoter region of chromatin accessibility and at the transcriptional level. It was reported that GSK-J4, another histone demethylase inhibitor, significantly reduced the expression of DNA DSB repair genes and DNA accessibility in diffuse intrinsic pontine glioma cells [15]. The repair of radiation-induced DNA damage in tumor cells affects resistance to radiation. A number of active chromatin processes to promote chromatin expansion for DNA repair have been proposed [32, 33]. Of these, H3K9 and H3K36 was reported to closely linked with DNA DSB repair [34]. However, there are also reports demonstrated chromatin condensation was integral to DNA damage response (DDR) signaling [35]. In addition, previous study reported that chromatin compaction protected genomic DNA from radiation damage and found that the frequency of radiation-induced DSBs in compact chromatin was 5–50-fold lower than in decondensed chromatin [36].

We also found that telomere maintenance was disrupted after IOX1 treatment. Telomeres prevent DSBs at the ends and prevent chromosome fusion [25]. Telomere are favorable targets for the sustained DNA damage response to radiation damage, and consider as a universal anticancer target in tumor cells [37]. Our results showed that radiation-induced telomeric damage repair was slowed down after IOX1 treatment. Transcriptomic data indicated that IOX1-induced downregulation of transcription levels of genes involved in maintaining telomere stability. Among them, PIF1 and TERT were found to be significantly downregulated at the chromatin accessibility level. TERT, as the catalytic subunit of telomerase, has been proven to play a protective effect on telomeres and could enhance the radiation resistance of cells [38]. PIF1 family helicases are evolutionarily conserved in prokaryotes and eukaryotes which could unwind the replication fork barriers such as G-tetraploid DNA, rDNA, and R-loop to promote replication. Studies have found that PIF1 can promote cell proliferation and inhibit apoptosis in cervical cancer [39]. Here, we found that PIF1 was highly expressed in multiple cancers compared to normal tissues, including lung cancer, suggesting that PIF1 may play a crucial promoting role in lung cancer development. Indeed, depletion of PIF1 delayed the repair of telomere damage and enhanced radiation-induced NSCLC apoptosis, suggesting that PIF1 is an important target for radiosensitivity. Furthermore, our comprehensive analysis, encompassing changes in *PIF1* chromatin accessibility, transcription factors binding to enhancers, and motif enrichment, suggested the involvement of MAZ in the transcriptional regulation of *PIF1* following IOX1 treatment. MAZ, a transcription factor, has been shown to exhibit aberrant expression in various tumor types, playing a role in the regulation of tumor cell proliferation, migration, apoptosis, and autophagy [40]. In lung cancer, MAZ promotes cisplatin resistance in lung adenocarcinoma cells by inhibiting DNA damage [30]. In this study, we observed a decrease in the enrichment of MAZ in the distal intergenic region of *PIF1* following IOX1 treatment to result the suppression of *PIF1* expression. Furthermore, the protein expression of MAZ was downregulated after IOX1 treatment, suggesting that IOX1 affected *PIF1* expression through direct alterations in chromatin accessibility of *PIF1* gene, possibly through indirect regulation by MAZ expression. We additionally showed that the inhibition of the *MAZ* gene enhanced the radiosensitivity of A549 cells, in line with the impact of low *PIF1* gene expression. This indicates that MAZ may act as a repressive factor in the regulation of radiosensitivity through chromatin accessibility.

In summary, our findings revealed that the demethylase inhibitor IOX1 played a crucial role in mediating the radiosensitivity of NSCLC by regulating chromatin accessibility. This effect was attributed to the changes of chromatin accessibility in inhibition DNA repair efficiency and disruption of telomere maintenance. Moreover, we found that PIF1 was involved in the enhancement of radiosensitivity of NSCLC whose expression levels were elevated in NSCLC, suggesting its potential application as a target for radiotherapy in NSCLC. Mechanistically, transcription factor MAZ promoted the reprogramming of *PIF1* and positively regulated its expression, which in turn impacted radiation-induced telomere dysfunction and apoptosis. This study provides new insights into the regulation of radiosensitivity at the chromatin level and provides a new target for radiosensitization of lung cancer.

### Supplementary information

Supplentary Methods

Supplementary Table 1

Supplementary Table 2

Supplementary Table 3

Supplementary Figure Legend

Figure S1

Figure S2

Figure S3

Figure S4

Figure S5

Figure S6

Figure S7

WB-Raw data

reproducibility checklist

## Data Availability

Genomic analyses were conducted using R unless otherwise stated. The sequences data reported in this study was archived in the Sequence Read Archive (SRA) with the accession number PRJNA952214. Other data that support the findings of this study are available from the corresponding author upon reasonable request.

## References

[1] Flavahan WA, Gaskell E, Bernstein BE (2017). Epigenetic plasticity and the hallmarks of cancer. Science.

[2] Zhao S, Allis CD, Wang GG (2021). The language of chromatin modification in human cancers. Nat Rev Cancer.

[3] Tome-Garcia J, Erfani P, Nudelman G, Tsankov AM, Katsyv I, Tejero R (2018). Analysis of chromatin accessibility uncovers TEAD1 as a regulator of migration in human glioblastoma. Nat Commun.

[4] Denny SK, Yang D, Chuang CH, Brady JJ, Lim JS, Gruner BM (2016). Nfib promotes metastasis through a widespread increase in chromatin accessibility. Cell.

[5] Nin DS, Wujanto C, Tan TZ, Lim D, Damen JMA, Wu KY (2021). GAGE mediates radio resistance in cervical cancers via the regulation of chromatin accessibility. Cell Rep.

[6] Corces MR, Granja JM, Shams S, Louie BH, Seoane JA, Zhou W (2018). The chromatin accessibility landscape of primary human cancers. Science.

[7] Sanghi A, Gruber JJ, Metwally A, Jiang L, Reynolds W, Sunwoo J (2021). Chromatin accessibility associates with protein-RNA correlation in human cancer. Nat Commun.

[8] Guler GD, Tindell CA, Pitti R, Wilson C, Nichols K, KaiWai Cheung T (2017). Repression of stress-induced LINE-1 expression protects cancer cell subpopulations from lethal drug exposure. Cancer Cell.

[9] Qu K, Zaba LC, Satpathy AT, Giresi PG, Li R, Jin Y (2017). Chromatin accessibility landscape of cutaneous T cell lymphoma and dynamic response to HDAC inhibitors. Cancer Cell.

[10] Suzuki S, Kato H, Suzuki Y, Chikashige Y, Hiraoka Y, Kimura H (2016). Histone H3K36 trimethylation is essential for multiple silencing mechanisms in fission yeast. Nucleic Acids Res.

[11] Li J, Bergmann L, Rafael de Almeida A, Webb KM, Gogol MM, Voigt P (2022). H3K36 methylation and DNA-binding both promote Ioc4 recruitment and Isw1b remodeler function. Nucleic Acids Res.

[12] Liscovitch-Brauer N, Montalbano A, Deng J, Méndez-Mancilla A, Wessels HH, Moss NG (2021). Profiling the genetic determinants of chromatin accessibility with scalable single-cell CRISPR screens. Nat Biotechnol.

[13] Hancock RL, Dunne K, Walport LJ, Flashman E, Kawamura A (2015). Epigenetic regulation by histone demethylases in hypoxia. Epigenomics.

[14] Macedo-Silva C, Miranda-Goncalves V, Lameirinhas A, Lencart J, Pereira A, Lobo J (2020). JmjC-KDMs KDM3A and KDM6B modulate radioresistance under hypoxic conditions in esophageal squamous cell carcinoma. Cell Death Dis.

[15] Katagi H, Louis N, Unruh D, Sasaki T, He X, Zhang A (2019). Radiosensitization by histone H3 demethylase inhibition in diffuse intrinsic pontine glioma. Clin Cancer Res.

[16] Herbst RS, Morgensztern D, Boshoff C (2018). The biology and management of non-small cell lung cancer. Nature.

[17] Szczepanski AP, Tsuboyama N, Watanabe J, Hashizume R, Zhao Z, Wang L (2022). POU2AF2/C11orf53 functions as a coactivator of POU2F3 by maintaining chromatin accessibility and enhancer activity. Sci Adv.

[18] Liu J, Zhao Z, Qiu N, Zhou Q, Wang G, Jiang H (2021). Co-delivery of IOX1 and doxorubicin for antibody-independent cancer chemo-immunotherapy. Nat Commun.

[19] Jie X, Fong WP, Zhou R, Zhao Y, Zhao Y, Meng R (2021). USP9X-mediated KDM4C deubiquitination promotes lung cancer radioresistance by epigenetically inducing TGF-β2 transcription. Cell Death Differ.

[20] Li S, Wang H, Jehi S, Li J, Liu S, Wang Z (2021). PIF1 helicase promotes break-induced replication in mammalian cells. EMBO J.

[21] Wang L, Chang J, Varghese D, Dellinger M, Kumar S, Best AM (2013). A small molecule modulates Jumonji histone demethylase activity and selectively inhibits cancer growth. Nat Commun.

[22] Taguchi J, Shibata H, Kabata M, Kato M, Fukuda K, Tanaka A (2021). DMRT1-mediated reprogramming drives development of cancer resembling human germ cell tumors with features of totipotency. Nat Commun.

[23] Lu B, Wei J, Zhou H, Chen J, Li Y, Ye L (2022). Histone H3K36me2 demethylase KDM2A promotes bladder cancer progression through epigenetically silencing RARRES3. Cell Death Dis.

[24] Jalal N, Haq S, Anwar N, Nazeer S, Saeed U (2014). Radiation induced bystander effect and DNA damage. J Cancer Res Ther.

[25] Ayouaz A, Raynaud C, Heride C, Revaud D, Sabatier L (2008). Telomeres: hallmarks of radiosensitivity. Biochimie.

[26] Berardinelli F, Coluzzi E, Sgura A, Antoccia A (2017). Targeting telomerase and telomeres to enhance ionizing radiation effects in in vitro and in vivo cancer models. Mutat Res Rev Mutat Res.

[27] Muellner J, Schmidt KH (2020). Yeast genome maintenance by the multifunctional PIF1 DNA helicase family. Genes.

[28] Jolma A, Yin Y, Nitta KR, Dave K, Popov A, Taipale M (2015). DNA-dependent formation of transcription factor pairs alters their binding specificity. Nature.

[29] Pastor WA, Liu W, Chen D, Ho J, Kim R, Hunt TJ (2018). TFAP2C regulates transcription in human naive pluripotency by opening enhancers. Nat Cell Biol.

[30] Wang T, Zhu X, Wang K, Li J, Hu X, Lin P (2023). Transcriptional factor MAZ promotes cisplatin-induced DNA damage repair in lung adenocarcinoma by regulating NEIL3. Pulm Pharm Ther.

[31] Liu D, Zhao Z, She Y, Zhang L, Chen X, Ma L (2022). TRIM14 inhibits OPTN-mediated autophagic degradation of KDM4D to epigenetically regulate inflammation. Proc Natl Acad Sci USA.

[32] Ziv Y, Bielopolski D, Galanty Y, Lukas C, Taya Y, Schultz DC (2006). Chromatin relaxation in response to DNA double-strand breaks is modulated by a novel ATM- and KAP-1 dependent pathway. Nat Cell Biol.

[33] Floyd SR, Pacold ME, Huang Q, Clarke SM, Lam FC, Cannell IG (2013). The bromodomain protein Brd4 insulates chromatin from DNA damage signalling. Nature.

[34] Ayrapetov MK, Gursoy-Yuzugullu O, Xu C, Xu Y, Price BD (2014). DNA double-strand breaks promote methylation of histone H3 on lysine 9 and transient formation of repressive chromatin. Proc Natl Acad Sci USA.

[35] Burgess RC, Burman B, Kruhlak MJ, Misteli T (2014). Activation of DNA damage response signaling by condensed chromatin. Cell Rep.

[36] Takata H, Hanafusa T, Mori T, Shimura M, Iida Y, Ishikawa K (2013). Chromatin compaction protects genomic DNA from radiation damage. PLoS ONE.

[37] Bejarano L, Bosso G, Louzame J, Serrano R, Gómez-Casero E, Martínez-Torrecuadrada J (2019). Multiple cancer pathways regulate telomere protection. EMBO Mol Med.

[38] Li Z, Zhang Y, Sui S, Hua Y, Zhao A, Tian X (2020). Targeting HMGB3/hTERT axis for radioresistance in cervical cancer. J Exp Clin Cancer Res.

[39] Wang J, Zhu X, Ying P, Zhu Y (2020). PIF1 affects the proliferation and apoptosis of cervical cancer cells by influencing TERT. Cancer Manag Res.

[40] Wang M, Yang X, Meng Y, Jin Z, Cao J, Xiong L (2023). Comprehensive analysis of the tumor-promoting effect and immune infiltration correlation MAZ from pan-cancer to hepatocellular carcinoma. Int Immunopharmacol.

