# Supplementary material for: Inhibition of demethylase by IOX1 modulates chromatin accessibility to enhance NSCLC radiation sensitivity through attenuated PIF1
Source: Cell Death Dis. 2023 Dec 12;14(12):817. doi: 10.1038/s41419-023-06346-2 (PMC10716120; doi:10.1038/s41419-023-06346-2)
Supplement: Supplementary file 1 — Supplentary Methods [file 41419_2023_6346_MOESM1_ESM.doc]

**Supporting Information for**

**Inhibition of demethylase by IOX1 modulates chromatin accessibility to enhance NSCLC radiation sensitivity through attenuated PIF1**

Qian Li ^1^, Kexin Qin ^2^, Yushan Tian ^3^, Biao Chen ^2^, Guoping Zhao ^1^, Shengmin Xu ^2^*, Lijun Wu ^1, 2^*

^1^School of Environmental Science and Optoelectronic Technology, University of Science and Technology of China, Hefei, Anhui, 230026, PR China

^2^Information Materials and Intelligent Sensing Laboratory of Anhui Province, Institutes of Physical Science and Information Technology, Anhui University, Hefei, Anhui, 230601, PR China

^3^China National Tobacco Quality Supervision and Test Center, Zhengzhou, Henan, 450001, PR China

* Corresponding author.

** Corresponding authors at: Information Materials and Intelligent Sensing Laboratory of Anhui Province, Institutes of Physical Science and Information Technology, Anhui University, Hefei, Anhui 230601, China. E-mail addresses: shmxu@mail.ustc.edu.cn (S. M. Xu), and [ljw@ipp.ac.cn](mailto:ljw@ipp.ac.cn) (L. J. Wu)

The authors declare that there are no competing interests.

**Supplementary Methods**

**1.1 Reagents and Antibodies**

5-Carboxy-8-hydroxyquinoline (IOX1) was purchased from Cayman Chemical (Cayman, MI, USA), and dissolved in dimethyl sulfoxide (DMSO) (Sango Biotech, Shanghai, China) to a 100 mM stock solution. The following antibodies were used in this study: mouse-anti-β-actin (Trans Gen Biotech, TA-09, Beijing, China), mouse-anti-PIF1 (Santa Cruz, 48377, CA, USA), mouse-anti-γH2AX (Millipore, 05-636, MA, USA), rabbit-anti-H3K9me3 (Abcam, 8898, MA, USA), rabbit-anti-H3K36me3 (Cell Signaling Technology, 4909T, MA, USA), mouse-anti-MAZ (Proteintech, 39936, Wuhan, China) and rabbit-cleaved-caspase3 (Cell Signaling Technology, 9664, MA, USA). Goat anti-rabbit IgG horseradish peroxidase (HRP) secondary antibody (12-348) and goat anti-mouse IgG HRP secondary antibody (12-349) were obtained from Sigma-Aldrich (Sigma-Aldrich, CA, USA). The secondary antibodies Alexa Fluor 488-labeled goat anti-rabbit IgG (A0423) and Alexa Fluor 488-labeled goat anti-mouse IgG (A0428) used for immunofluorescence were purchased from Beyotime Inc. (Beyotime, Shanghai, China).

**1.****2 Immunofluorescence staining, telo-FISH assay and immunohistochemistry**

For immunofluorescence staining, cells were fixed with 4% paraformaldehyde (PFA) in PBS for 20 min at room temperature. Fixed cells were then permeabilized with 0.5% Triton-X100 (Sangon, China) in 1×PBS (PBST) for 30 min. Cells were blocked with 5% BSA (Sangon, China) in 0.1% PBST for 1 h. Then, cells were subsequently incubated with primary antibody overnight at 4°C (Mouse-anti-γH2AX, 1:1000; Rabbit-anti-H3K9me3, 1:1000; Mouse-anti-H3K36me3, 1:1000). Next day, cells were washed with 0.1% PBST 3×5 mins, then incubated with Alexa Fluor 488-labeled goat anti-mouse IgG or Alexa Fluor 488-labeled goat anti-rabbit IgG for 1 h in the dark. Nuclei were stained with Hoechst 33342 (Beyotime, China). Images were acquired with a Zeiss LSM 710 microscope using 63×oil lens or Leica DMI 4000B microscope using 20× or 100×oil lens and analyzed by ZEN3.0 or LAX S.

Telo-FISH was performed with telomeric peptide nucleic acid (PNA) probe after γH2AX immunostaining as described above. After finished γH2AX immunostaining with Alexa Fluor 488-labeled goat anti-mouse IgG, postfix the secondary antibody to primary antibody in 4% PFA for 10 min at room temperature. Cells were hybridized with 0.5 μg/ml telomeric PNA probe (TelC-Cy3-CCCTAACCCTAACCCTAA) in hybridization buffer (70% formamide, 0.06×SSC, 0.001% BSA) at 90℃ for 3 min, followed by incubating in the dark for overnight in a humidified chamber at 4℃. Cells were washed with wash solution I (70% formamide, 10 mM Tris-HCl, pH 7.5, 0.1% Tween-20, 0.1% BAS) for 3×15 mins and wash solution II (150 mM NaCl, 50 mM Tris-HCl, pH=7.5, 0.1% Tween-20, 0.1% BAS) for 3×15 mins. Then, nuclei were stained with Hoechst 33342. Images were acquired with a Zeiss LSM 710 microscope using 63×oil lens and analyzed by ZEN3.0.

For the immunohistochemistry studies, following indicated treatment, A549 tumor was harvested and fixed in 4% PFA for 1 h. After embedded in paraffin, the 3-to-4-μm-thick histological sections were cut and tissue sections were stained with γH2AX as well as cleaved-caspase3 antibody. Images were acquired using a fluorescence microscope (Leica DMI4000 B).

**1.3 Cell proliferation and cell viability assay**

Proliferation was assessed by EdU incorporation. Cells were incubated with 10 μM EdU for 2 h and then fixed with 4% PFA for 10 min. Cell membrane was permeated with 0.5% PBST for 30 min. The following staining was performed using the BeyoClick™ kit (Beyotime, China) based on the manufacturer’s protocol. Nuclei were stained with Hoechst 33342 and the images was acquired by fluorescence microscope (Leica DMI4000B).

CCK8 assay was used to determine cell viability. Briefly, cells were seeded at 8000 cells/well in 96-well plates and incubated overnight at 37°C. After indicated treatment, 10% CCK8 solution was added to the cells and incubated for another 2 h at 37°C away from light. The absorbance was measured with a microplate reader (Spectra Max i3x) at 450 nm. Three replicate wells were included in each analysis.

**1.4 Western blot**

Proteins were extracted from the cells using RIPA lysate buffer (Beyotime, China) and quantified by BCA kit (ThermoFisher Scientific, MA, USA). The samples were resolved by SDS-page gels and then transferred to poly-vinylidene difluoride (PVDF) membranes (Roche, IN, USA). After blocked with 5% BSA at room temperature for 1 h, the membranes were probed with the corresponding primary antibodies overnight at 4°C. Next day, the second HRP-linked anti-rabbit or anti-mouse IgG antibody was incubated at room temperature for 1 h. Finally, the immunoblots were detected by an enhanced chemiluminescence assay (Biosharp, Anhui, China). Densitometry was analyzed by ImageJ V1.8.0 (NIH, MD, USA). β-actin run on the same blot were used as the loading controls.

**1.5 Quantitative real time PCR (qRT-PCR)**

Total RNA was extracted from treated cells using Trizol reagent (Ambion Life Technologies, CA, USA). First-strand complementary DNA (cDNA) was obtained by reverse transcription using the TransScript One-step gDNA Removal and cDNA Synthesis SuperMix (Trans Gen Biotech, China). qRT-PCR was performed with 500 ng cDNA using TransStart® Top Green qPCR SuperMix (Trans Gen Biotech, China) in a total volume of 20 μl. The sequences of primers used are listed in Table S2. qRT-PCR was carried out with the set procedure of 95°C for 30 s and 40 cycles of 95°C for 5 s, 60°C for 15 s and 72°C for 10 s on a Roche LC96 instrument (Roche Diagnostics, Mannheim, Germany). All genes were normalized to *GAPDH* expression levels. Relative expression was calculated by comparative Δ ΔCt method.

**1.6 CUT & Tag-qPCR**

A549 cells were cultured in 60-mm petri dishes and treated with DMSO or 40 μM IOX1 for 48 h. CUT & Tag analysis was carried out using NovoNGS®CUT&Tag 4.0 High-Sensitivity Kit (Novoprotein, Suzhou, China) according to the manufacture’s recommended protocol. Anti-MAZ antibody was used to pull down DNA-protein complexes, and rabbit IgG (Santa Cruz, USA) was used as a negative control. The amount of purified DNA was quantified by qRT-PCR and normalized to *GAPDH*. The sequence of primers used are listed in Table S2.
