# Supplementary material for: Inhibition of demethylase by IOX1 modulates chromatin accessibility to enhance NSCLC radiation sensitivity through attenuated PIF1
Source: Cell Death Dis. 2023 Dec 12;14(12):817. doi: 10.1038/s41419-023-06346-2 (PMC10716120; doi:10.1038/s41419-023-06346-2)
Supplement: Supplementary file 2 — Supplementary Table 1 [file 41419_2023_6346_MOESM2_ESM.docx]

**Supplementary Table 1**

| Table. S1 Sequences of shRNA and siRNAs | |
| --- | --- |
| Name | sequence (5'-3') |
| *PIF1*-shRNA | GAAGACAGGUGCTCCGGAAGC |
| *MAZ*-siRNA-1 | AGGAGUUCAAGAACGGCUACA |
| *MAZ*-siRNA-2 | GUCAGACAAGUGCACUCAACA |
