# Supplementary material for: Inhibition of demethylase by IOX1 modulates chromatin accessibility to enhance NSCLC radiation sensitivity through attenuated PIF1
Source: Cell Death Dis. 2023 Dec 12;14(12):817. doi: 10.1038/s41419-023-06346-2 (PMC10716120; doi:10.1038/s41419-023-06346-2)
Supplement: Supplementary file 3 — Supplementary Table 2 [file 41419_2023_6346_MOESM3_ESM.docx]

**Supplementary Table 2**

| Table. S2-1 Primers used in real time PCR analysis | | | | |
| --- | --- | --- | --- | --- |
| Gene* | Forward sequence (5'-3') | | Reverse sequence (5'-3') | |
| *RAD51D* | CCACAGGATCAAGACAGTGGT | | AGGATGGCAGTGGAGGTCTT | |
| *HES1* | TGTCAACACGACACCGGATAA | | ATGCCGCGAGCTATCTTTC | |
| *POLD1* | CTGTTTGAAGCGGCAGGATG | | GGATCTATGGCTGATGGTGGG | |
| *MCM5* | AGCATTCGTAGCCTGAAGTCG | | CGGCACTGGATAGAGATGCG | |
| *PIF1* | GTCGGCACAAGTGAGTGTCT | | GCTCCGAGTCCTCATATTCCC | |
| *MAZ* | CCGGATCACCTCAACAGTCA | | AAAGCTGCCTCACATTTCTCAC | |
| *GAPDH* | ACCCAGAAGACTGTGGATGG | | CACATTGGGGGTAGGAACA | |
|  |  | | |  |
| Table. S2-2 Primers used in CUT-Tag-qPCR analysis | | | | |
| Fragment | Forward sequence (5'-3') | Reverse sequence (5'-3') | | |
| 1 | CTTTATAGCGCCGACCA | GCACAACCAGATAGAACCG | | |
| 2 | ACAGGTGGGGCTGATGAGTT | AGCCAGCATCCGGTTCTATC | | |

| Table. S2-3 Primers used in ATAC-qPCR analysis | | |
| --- | --- | --- |
| Fragment | Forward sequence (5'-3') | Reverse sequence (5'-3') |
| *PIF1* | CAGCGGGGATGGGATATTGG | AGGACTGGGTCTGGAACTCA |
| *TERT* | GAGGGGGTGAAATCGGGACT | GGGTGTTCAGGGGATGGTG |
