# Supplementary material for: Inhibition of demethylase by IOX1 modulates chromatin accessibility to enhance NSCLC radiation sensitivity through attenuated PIF1
Source: Cell Death Dis. 2023 Dec 12;14(12):817. doi: 10.1038/s41419-023-06346-2 (PMC10716120; doi:10.1038/s41419-023-06346-2)
Supplement: Supplementary file 4 — Supplementary Table 3 [file 41419_2023_6346_MOESM4_ESM.docx]

**Supplementary Table 3**

| Table. S3 Quality analysis of ATAC-seq data | | | | | | | |
| --- | --- | --- | --- | --- | --- | --- | --- |
| Sample | DMSO-1 | DMSO-2 | DMSO-3 | IOX1-1 | IOX1-2 | IOX1-3 | average |
| Raw reads | 109,165,230 | 82,767,353 | 79,597,140 | 197,548,619 | 126,557,429 | 192,139,857 | 131,295,938 |
| Raw bases(G) | 32.7496 | 24.8302 | 23.8791 | 59.2646 | 37.9672 | 57.642 | 39 |
| Clean reads | 109,073,914 | 82,662,799 | 79,575,009 | 197,433,657 | 126,488,293 | 191,985,889 | 131,203,260 |
| Clean bases(G) | 25.5447 | 19.6575 | 17.7015 | 45.3006 | 28.975 | 43.8588 | 30 |
| Effective rate(%) | 78 | 79.17 | 74.13 | 76.44 | 76.32 | 76.09 | 76.69166667 |
| Q20(%) | 98.29 | 98.41 | 98.68 | 96.94 | 97.51 | 98.26 | 98.015 |
| Q30(%) | 94.61 | 95.32 | 95.89 | 91.13 | 92.5 | 94.48 | 93.98833333 |
| Error rate(%) | 0.03 | 0.03 | 0.025 | 0.03 | 0.03 | 0.03 | 0.029166667 |
| GC ration(%) | 36.63 | 45.12 | 40.88 | 32.54 | 32.3 | 36.16 | 37.27166667 |
