# Supplementary material for: Inhibition of demethylase by IOX1 modulates chromatin accessibility to enhance NSCLC radiation sensitivity through attenuated PIF1
Source: Cell Death Dis. 2023 Dec 12;14(12):817. doi: 10.1038/s41419-023-06346-2 (PMC10716120; doi:10.1038/s41419-023-06346-2)
Supplement: Supplementary file 5 — Supplementary Figure Legend [file 41419_2023_6346_MOESM5_ESM.docx]

**Supplementary Figure Legend**

Figure S1. (A) The gene expression of histone demethylases (KDM4A, KDM4B, KDM2A) in clinical lung cancer and normal lung tissues were searched in the TCGA. (B) A549 cells were treated with different concentrations of IOX1 for 48 h. Cell viability was measured by CCK8 assay (top). A549 cells were treated with 40 μM of IOX1 for different times. And then the cell viability was measured by CCK8 assay (bottom). (C) Flow cytometry was used to detect the apoptosis of H1299, H1975 and HeLa cells in the IOX1-treated group and the control group at 48 h after 4 Gy γ-irradiation. Histogram represents the percentage of annexin V^+^PI^-^cells in early apoptosis and annexin V^+^PI^+^ in late apoptosis. The effect of IOX1 on A549 (D) and H1299 (E) cell proliferation after 4 Gy γ-irradiation was determined by EdU labeling. Red nuclei indicated EdU incorporation. Scale bar, 20 μm. The data are presented as the mean ± SD from three independent experiments. *P*-values were analyzed by Two-way analysis of variance (ANOVA) in multiple groups, **P*<0.05, ***P*<0.01.

Figure S2. Immunofluorescence in situ hybridization (FISH) with γH2AX antibody (Green) and telomere PNA probe (Red) was used to detect DNA damage and telomere dysfunction-induced foci (TIFs) in IOX1-treated and DMSO-treated control H1299 cells at indicated times after γ-irradiation. White arrows point to telomeric DNA damage. Scale bar, 2 μm. Quantification of γH2AX positive cells (top right) and TIF-positive cells (bottom right) in figure. Data were presented as the mean ± SD from three independent experiments. *P*-values were analyzed by Two-way analysis of variance (ANOVA) in multiple groups, **P*<0.05.

Figure S3. H3K9me3 and H3K36me3 expression levels in A549 cells after treatment with 40 μM of IOX1 for 48 h by western blot analysis (left) and immunofluorescence assay (right). Representative photos of H3K9me3 and H3K36me3 detection by immunofluorescence. Hoechst33342 staining marks nuclei. Scale bar, 2 μm.

Figure S4. (A) Correlation analysis of chromatin accessibility changes in three repeated samples after IOX1 treatment. (B) Heatmap depicting ATAC-seq enrichment of peaks near the accessible promoters (1.0 kb upstream TSS and downstream TES per gene, respectively) present in control and IOX1 treated cells. The whole genomic range was evaluated per sample. (C) Volcano plot of genes that are differentially expressed between the control and IOX1 treated cells. Red and green dots represent genes significantly up-regulated and downregulated in IOX1-treated group compared with control group (FDR<0.05), respectively. (D) Correlation analysis between chromatin accessibility in different functional regions of genes and the transcription levels with the treatment of IOX1.

Figure S5. (A) *PIF1* mRNA levels (left) and protein levels (right) in IOX1-treated A549 cells. (B) The relative PIF1 protein levels in A549 cells and (C) in H1299 cells expressing shRNA against *PIF1* were determined by western blot. The data are presented as the mean ± SD from three independent experiments. *P*-values were analyzed by Student’s t test between two groups. **P* < 0.05, ***P* < 0.01.

Figure S6. (A) Statistically significant MAZ transcription factor binding motifs enriched in ATAC peaks significantly downregulated after IOX1 treatment were calculated using HOMER software. (B) Expression level of *MAZ* gene after siRNA-mediated *MAZ* interference was determined by qRT-PCR. (C) Protein expression of MAZ after treated with IOX1 for 48 h in A549 cells was determined by western blot. (D) Protein expression of MAZ in three NSCLC cell lines (A549, H1299, H1975) and normal human fetal lung fibroblast MRC5 cells was determined by western blot. EdU (E) and CCK8 (F) assays were used to detect the influence of MAZ inhibition on radiation-induced proliferation activity in A549 cells. Red nuclei indicated EdU incorporation. Scale bar, 20 μm. The data are presented as the mean ± SD from three independent experiments. *P*-values were analyzed by Student’s t test between two groups and two-way analysis of variance (ANOVA) in multiple groups. **P* < 0.05, ***P* < 0.01，****P* < 0.001.

Figure S7. (A) Cell viability of A549-shPIF1 and (B) H1299-shPIF1 at 48 h post-4 Gy γ-irradiation was assessed by CCK 8 assay. (C) Overexpression of PIF1 protein in A549-PIF1 cells and (D) H1299-PIF1 cells was confirmed by western blot. (E) Proliferation activity in A549-PIF1 and (F) H1299-PIF1 treated with IOX1 after 4 Gy γ-irradiation was measured by EdU assay. Scale bar, 20 μm. The data are presented as the mean ± SD from three independent experiments. *P*-values were analyzed by Student’s t test between two groups and two-way analysis of variance (ANOVA) in multiple groups. **P* < 0.05, ***P* < 0.01.
