# Supplementary figures and images for: Inhibition of demethylase by IOX1 modulates chromatin accessibility to enhance NSCLC radiation sensitivity through attenuated PIF1
Source: Cell Death Dis. 2023 Dec 12;14(12):817. doi: 10.1038/s41419-023-06346-2 (PMC10716120; doi:10.1038/s41419-023-06346-2)

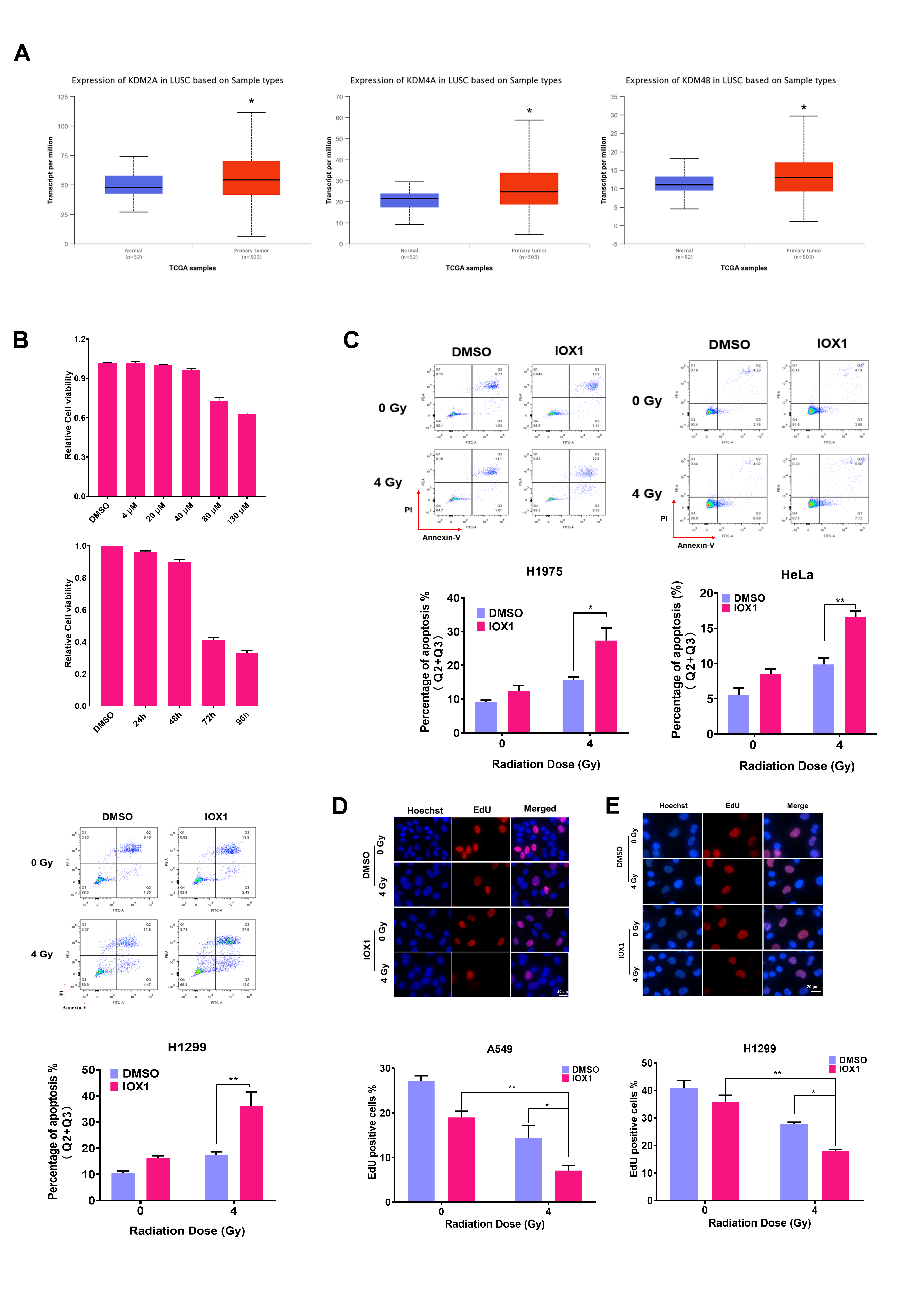

Supplement: Supplementary file 6 — Figure S1 [file 41419_2023_6346_MOESM6_ESM.tif]

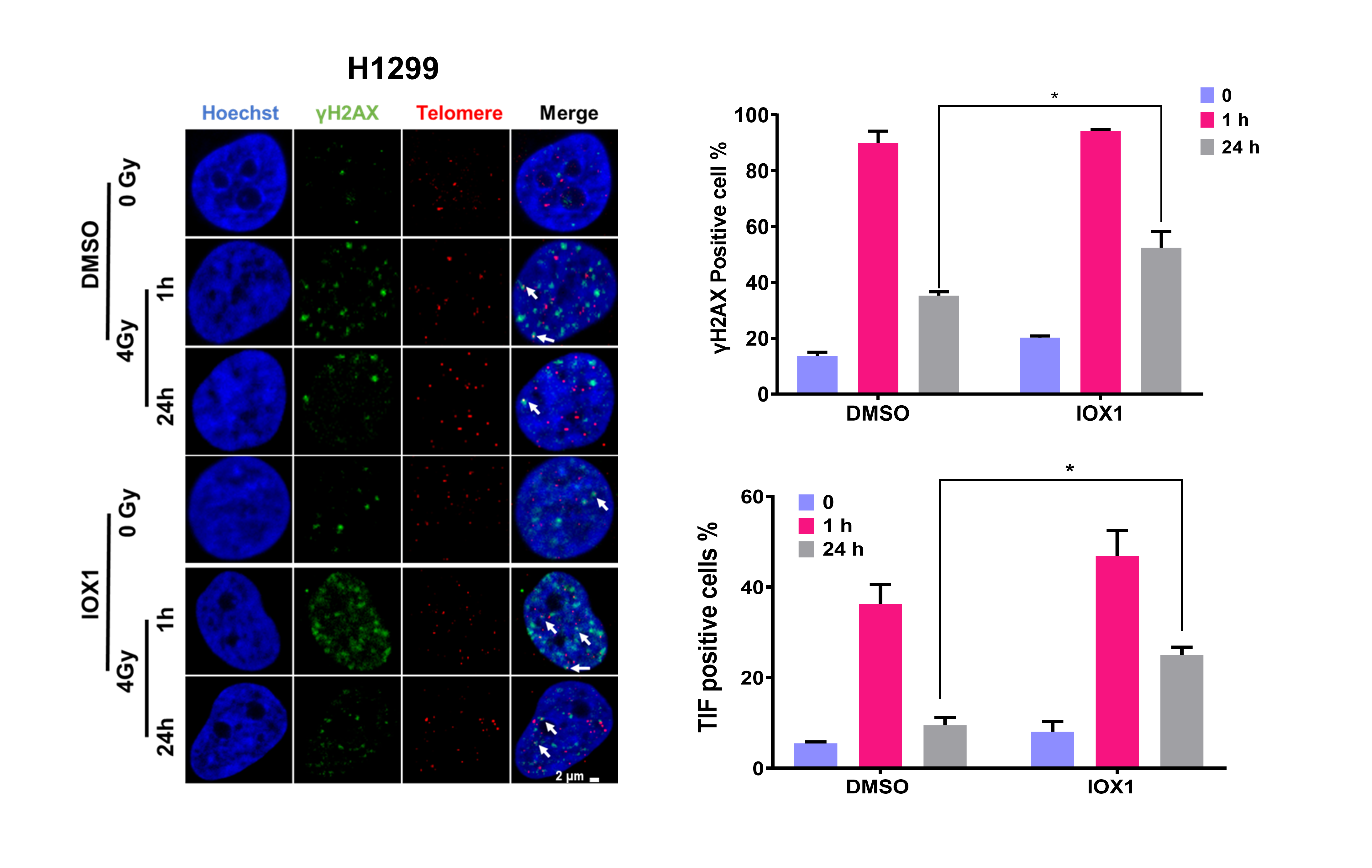

Supplement: Supplementary file 7 — Figure S2 [file 41419_2023_6346_MOESM7_ESM.tif]

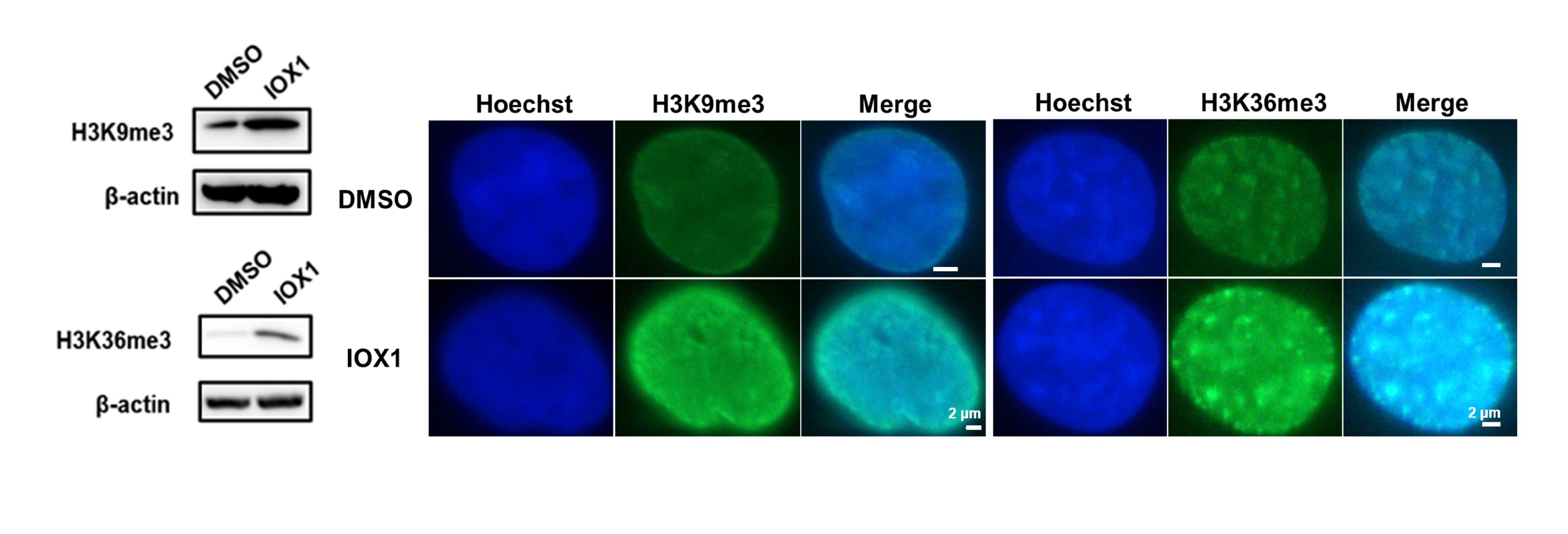

Supplement: Supplementary file 8 — Figure S3 [file 41419_2023_6346_MOESM8_ESM.tif]

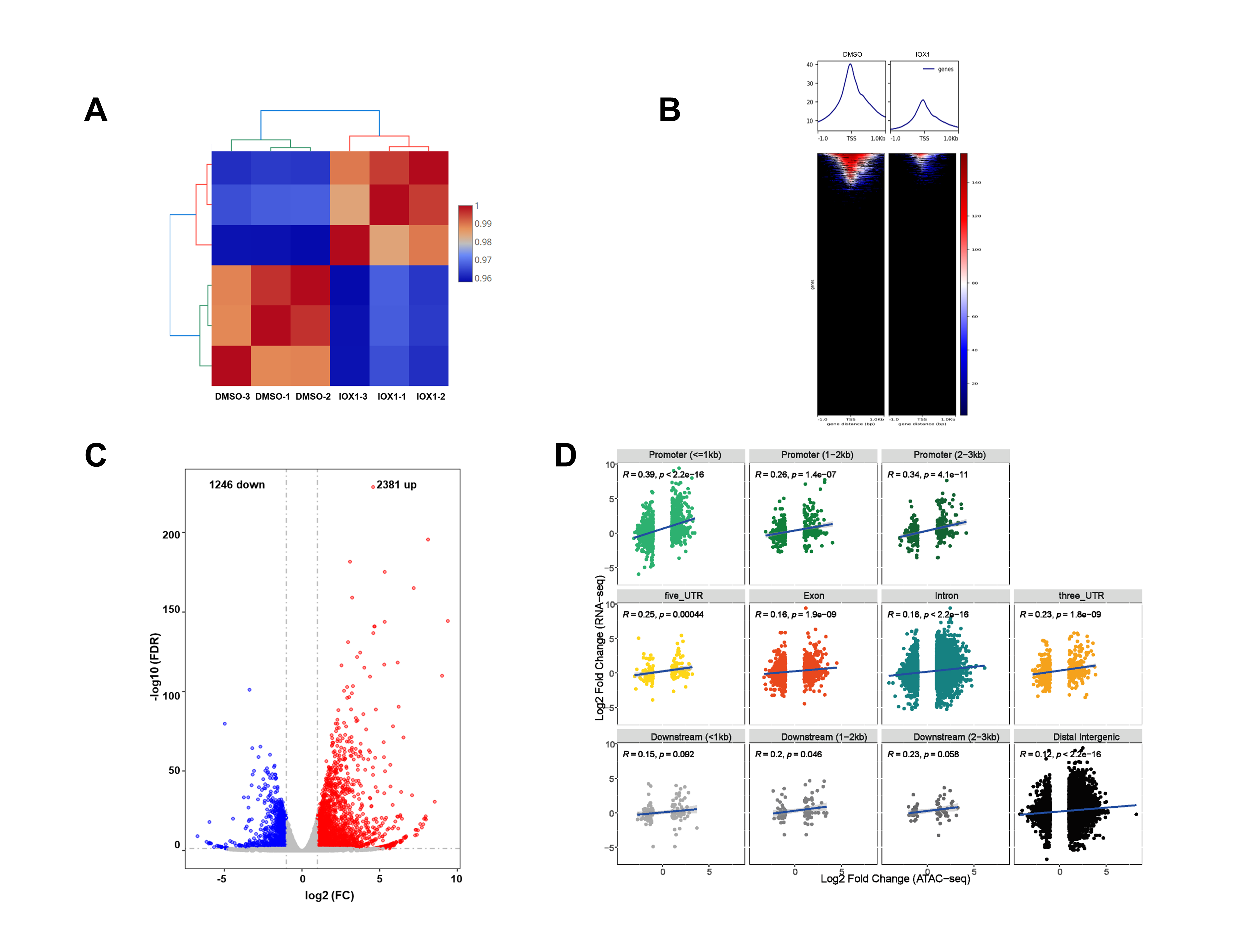

Supplement: Supplementary file 9 — Figure S4 [file 41419_2023_6346_MOESM9_ESM.tif]

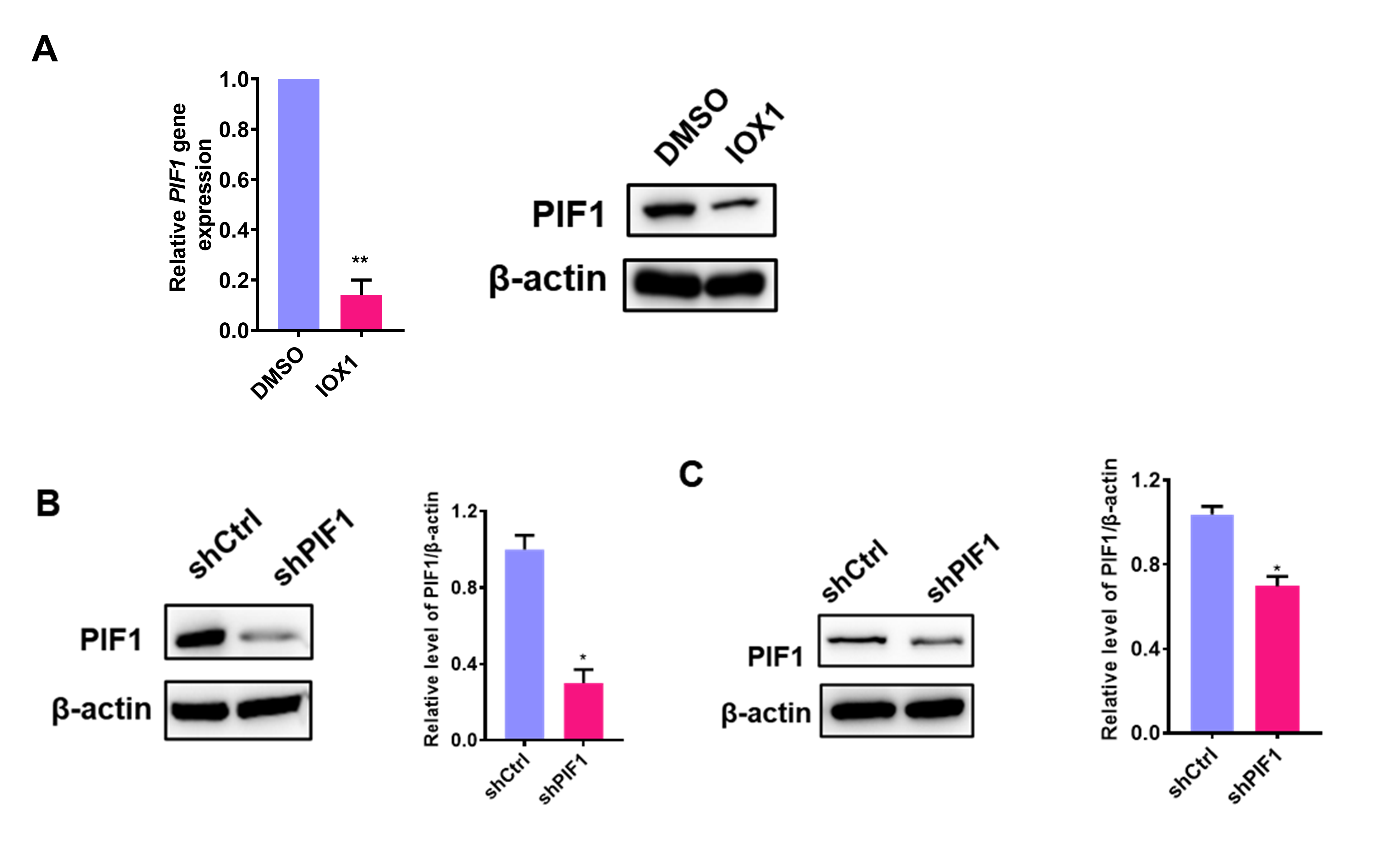

Supplement: Supplementary file 10 — Figure S5 [file 41419_2023_6346_MOESM10_ESM.tif]

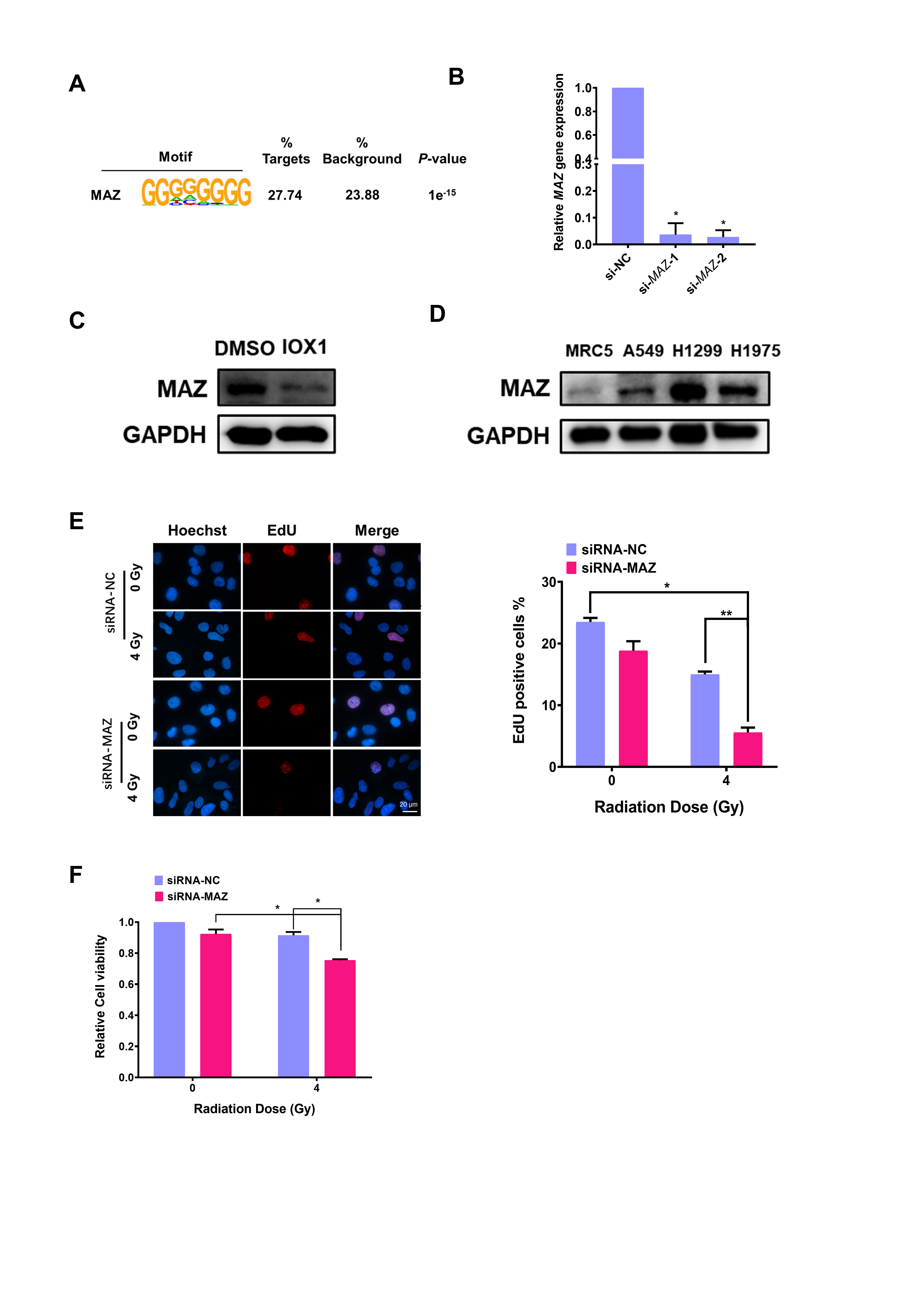

Supplement: Supplementary file 11 — Figure S6 [file 41419_2023_6346_MOESM11_ESM.tif]

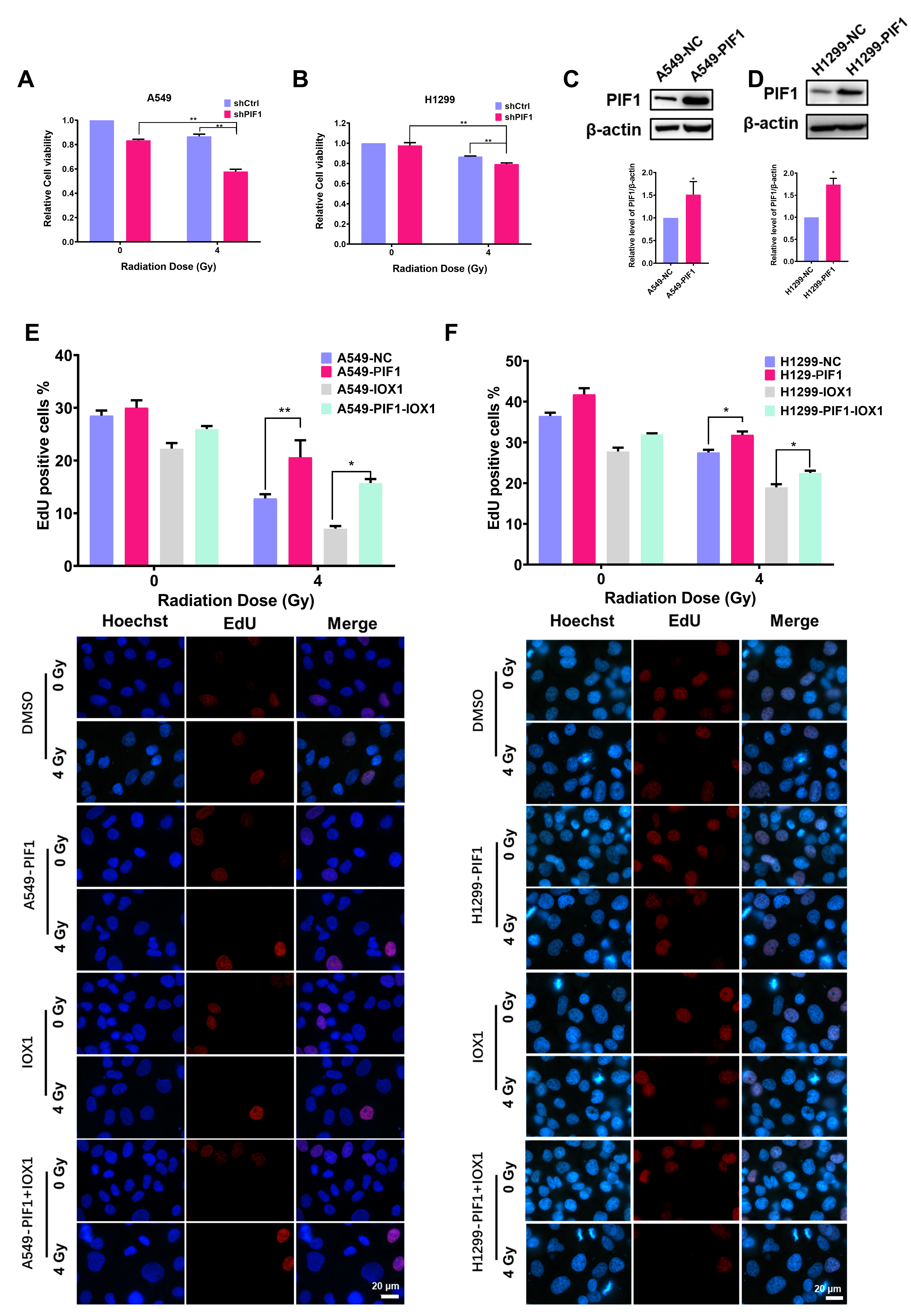

Supplement: Supplementary file 12 — Figure S7 [file 41419_2023_6346_MOESM12_ESM.tif]
