# Supplementary material for: Inhibition of demethylase by IOX1 modulates chromatin accessibility to enhance NSCLC radiation sensitivity through attenuated PIF1
Source: Cell Death Dis. 2023 Dec 12;14(12):817. doi: 10.1038/s41419-023-06346-2 (PMC10716120; doi:10.1038/s41419-023-06346-2)
Supplement: Supplementary file 13 — WB-Raw data [file 41419_2023_6346_MOESM13_ESM.docx]

**



**

**Fig. 4B-PIF1 Fig. 4B-β-actin**

**



**

**Fig. S3-H3K9me3 Fig. S3-β-actin-1**

**



**

**Fig. S3- H3K36me3 Fig. S3-****β-actin-2**







**Fig. S5A-PIF1 Fig. S5A-β-actin**

**



**

**Fig. S5B-PIF1 Fig. S5B-β-actin**

**



**

**Fig. S5C-PIF1 Fig. S5C-β-actin**

**



**

**Fig. S6C-MAZ Fig. S6C-GAPDH**

**



**

**Fig. S6D-MAZ Fig. S6D-GAPDH**







**Fig. S7C-A549-PIF1 Fig. S7C-A549-β-actin**







**Fig. S7D-H1299-PIF1 Fig. S7D-H1299-β-actin**
